# The Type I Interferon Pathway Is Upregulated in the Cutaneous Lesions and Blood of Multibacillary Leprosy Patients With Erythema Nodosum Leprosum

**DOI:** 10.3389/fmed.2022.899998

**Published:** 2022-06-06

**Authors:** Thabatta Leal Silveira Andrezo Rosa, Mayara Abud Mendes, Natasha Ribeiro Cardoso Linhares, Thais Fernanda Rodrigues, André Alves Dias, Thyago Leal-Calvo, Mariana Gandini, Helen Ferreira, Fabrício da Mota Ramalho Costa, Anna Maria Sales, Thaís Porto Amadeu, Veronica Schmitz, Roberta Olmo Pinheiro, Luciana Silva Rodrigues, Milton Ozório Moraes, Maria Cristina Vidal Pessolani

**Affiliations:** ^1^Laboratory of Cellular Microbiology, Oswaldo Cruz Institute, Oswaldo Cruz Foundation, Rio de Janeiro, Brazil; ^2^Laboratory of Leprosy, Oswaldo Cruz Institute, Oswaldo Cruz Foundation, Rio de Janeiro, Brazil; ^3^Laboratory of Immunopathology, Medical Science Faculty, Rio de Janeiro State University, Rio de Janeiro, Brazil

**Keywords:** immunopathogenesis, leprosy reaction, type I interferons, plasmacytoid dendritic cells, erythema nodosum leprosum

## Abstract

In leprosy patients, acute inflammatory episodes, known as erythema nodosum leprosum (ENL), are responsible for high morbidity and tissue damage that occur during the course of *Mycobacterium leprae* infection. In a previous study, we showed evidence implicating DNA-sensing *via* TLR9 as an important inflammatory pathway in ENL. A likely important consequence of TLR9 pathway activation is the production of type I interferons (IFN-I) by plasmacytoid dendritic cells (pDCs), also implicated in the pathogenesis of several chronic inflammatory diseases. In this study, we investigated whether the IFN-I pathway is activated during ENL. Blood samples and skin lesions from multibacillary patients diagnosed with ENL were collected and the expression of genes of the IFN-I pathway and interferon-stimulated genes were compared with samples collected from non-reactional multibacillary (NR) patients. Whole blood RNAseq analysis suggested higher activation of the IFN-I pathway in ENL patients, confirmed by RT-qPCR. Likewise, significantly higher mRNA levels of IFN-I-related genes were detected in ENL skin biopsies when compared to NR patient lesions. During thalidomide administration, the drug of choice for ENL treatment, a decrease in the mRNA and protein levels of some of these genes both in the skin and blood was observed. Indeed, *in vitro* assays showed that thalidomide was able to block the secretion of IFN-I by peripheral blood mononuclear cells in response to *M. leprae* sonicate or CpG-A, a TLR9 ligand. Finally, the decreased frequencies of peripheral pDCs in ENL patients, along with the higher TLR9 expression in ENL pDCs and the enrichment of CD123^+^ cells in ENL skin lesions, suggest the involvement of these cells as IFN-I producers in this type of reaction. Taken together, our data point to the involvement of the pDC/type I IFN pathway in the pathogenesis of ENL, opening new avenues in identifying biomarkers for early diagnosis and new therapeutic targets for the better management of this reactional episode.

## Introduction

Interferons constitute a broad class of cytokines, classified into types I, II, and III, performing multifaceted roles. Type I IFNs make up the largest family of interferons, which, in humans, is composed of thirteen IFN-α subtypes and one copy of IFN-β, IFNε, IFNκ, and IFNω each. IFN-α along with IFN-β are the most abundant and well characterized (1). Although best known for their overall antiviral activity, IFN-α and IFN-β are now recognized for their roles as modulators in the immune response against parasites, bacteria, and fungi (2, 3), as well as in the immunopathogenesis of a number of inflammatory and autoimmune diseases (4, 5). All type I IFNs bind to the same ubiquitously- expressed type I IFN receptor (IFNAR), composed of the IFNAR1 and IFNAR2 subunits (5). The type I interferons signaling pathway culminates in the induction of hundreds of interferon-stimulated genes (ISGs) that mediate antiviral effects and other biological activities, leading to the emergence of what is called an *interferon signature* (6).

The role of IFN-I in the pathogenesis of systemic lupus erythematosus (SLE) and other inflammatory diseases (7, 8), including the potential use of this pathway in controlling disease symptoms, has been extensively explored over the last few decades (9–11). It is now well established that type I interferons (most related studies have explored IFN-α/β functions) crucially affect different subsets of innate and adaptive immune cells that can exacerbate inflammation and lead to immunopathology and tissue damage (5, 12, 13). Increasing evidence also points to plasmacytoid dendritic cells (pDCs) as the major source of IFN-α/β in these diseases *via* the recognition of extracellular self-nucleic acids such as nucleic acid-immune complexes and extracellular neutrophil traps (NETs) by way of the toll-like receptors 7 (TLR7) and 9 (TLR9; 5, 9, 10, 14). The pathogenic role of pDCs-derived IFN-I has been especially emphasized in inflammatory diseases with cutaneous manifestations during which these cells accumulate in the skin lesions (15–18).

In about 40% of leprosy patients, acute inflammatory episodes, known as leprosy reactions, may occur before, during, or after multidrug therapy (MDT). These inflammatory episodes complicate the course of *Mycobacterium leprae* infection and are responsible for high morbidity and tissue damage (19, 20). Thus, the early diagnosis and treatment of leprosy reactions are highly relevant since these episodes exacerbate nerve damage and may also be a major cause of patient hospitalization and death. Most are classified as either type 1 reaction/reverse reaction (RR) or type 2 reaction/erythema nodosum leprosum (ENL). ENL only affects the multibacillary clinical forms: borderline lepromatous (BL) and lepromatous leprosy (LL; 20, 21). ENL is characterized by the sudden appearance of small, reddened skin nodules, in most cases accompanied by systemic symptoms like fever, malaise, iritis, arthritis, neuritis, and lymphadenitis, in addition to peripheral nerve impairment (20). The severity of ENL varies and is classified according to the clinical symptoms of the patient as mild, moderate, or severe (22). Frequently recurrent, it often requires long-term treatment with oral corticosteroids (20, 21, 23). However, an effective alternative drug is thalidomide. This medication has been shown to be extremely beneficial within a short period of time (24, 25). However, due to its teratogenic effects, its use is tightly restricted and only allowed in Brazil and a few other countries (23, 25).

In a previous report, evidence was presented that implicated DNA sensing *via* TLR9 as an important inflammatory pathway in ENL. Higher TLR9 expression levels in skin lesions and blood cells together with higher circulating levels of endogenous and *M. leprae*-derived TLR9 ligands were detected in these patients (26). In the present study, the hypothesis under review was that TLR9 activation by autologous and/or *M. leprae* DNA induces the production of type I interferons by pDCs, perhaps contributing to ENL immunopathogenesis in a way similar to what is observed in other inflammatory diseases with cutaneous manifestations.

## Materials and Methods

### Subjects and Clinical Specimens

The study population consisted of leprosy patients referred to the Souza Araujo Outpatient Clinic, a Reference Center for Leprosy Diagnosis and Treatment, Oswaldo Cruz Foundation (FIOCRUZ) in Rio de Janeiro, RJ, Brazil. Age- and ethnic-matched healthy volunteers were also recruited among the Leprosy Laboratory staff at the same institution to serve as healthy donors (HD). Each patient was clinically assessed by way of detailed clinical and dermatological examinations, after which WHO–recommended MDT for leprosy patients was administered. Bacteriological examinations of slit-skin smears were done to determine the bacilloscopic index. None of the participants displayed any infectious or chronic inflammatory co-morbidities such as HIV, syphilis, hepatitis, cancer, or diabetes. The present study was approved by the FIOCRUZ Committee for Ethics in Research (CAAE 56113716.5.0000.5248). Written informed consent was obtained from all participants prior to enrollment and sample collection.

Leprosy patients were diagnosed and categorized according to the Ridley and Jopling classification scale (27) as BL or LL. ENL diagnosis was primarily based on the occurrence of nodular skin lesions with or without fever and peripheral nerve pain and/or nerve dysfunction. Clinical samples were collected from three different groups of patients: (i) The NR group – BL/LL patients with no signs of leprosy reaction at diagnosis; (ii) The ENL group – patients at the onset of reaction and before thalidomide or corticosteroid administration, none of whom had been treated with corticosteroid and/or thalidomide for at least 4 months prior to recruitment; and (iii) The ENL_Thal_ patients recruited for a reevaluation at day 7 after starting thalidomide at 100–300 mg/day. Punch biopsies (6 mm in diameter) from active skin lesions, containing both epidermis and dermis, were obtained at diagnosis. These specimens were split in parts and later used for histopathological, molecular, and Western blot analyses. Whole blood samples were collected from leprosy patients and HD for *in vitro* stimulation assays and *ex vivo* analysis. The baseline characteristics of the patients enrolled in the study are shown in Supplementary Tables 1–5.

### RNAseq Analysis

Total RNA from 2.5 mL of fresh blood collected in the PAXgene Blood RNA Tube (Qiagen, Germany) was extracted using the PAXgene blood RNA kit (Qiagen), as instructed by the manufacturer. RNA quality was assessed *via* Agilent tapeStation 2200 (Agilent, United States), with the sole inclusion of RNAs with RIN > 8. A polyA-enriched complementary DNA (cDNA) library was obtained using the NEBNext Poly(A) mRNA magnetic isolation module (New England Bioscience, United States) and NEBNext Ultra II Directional RNA for Illumina kit (New England Bioscience). RNA sequencing was performed on the NextSeq550 Illumina platform with 75 paired-end cycles (Illumina, United States). The raw RNAseq dataset is readily available at Gene Expression Omnibus (GSE198609). For the initial read quality control, FastQC v.0.11.8^1^ and MultiQC v.1.9 (28) were utilized. To remove adapter and poly-X sequences and trim the first 10 bases at 5’-end, fastp v.0.21.0 was applied. Pre-processed reads were quantified against the human transcriptome (GRCh38p.12)^2^ with the Salmon v.1.4. pipeline *via* the quasi-mapping method with the –gcBias and –seqBias flags set and other default settings. Transcript quantification was summarized into ENSEMBLE genes with tximport v.1.12.0 (29) and biomaRt v.2.40.5 (30). Differential gene expression analysis was performed with DESeq2 v.1.24.0 (31); and *p*-values were adjusted by the Benjamini and Hochberg method to control the false discovery rate (FDR; 32). Fold-changes were moderated with the “ashr” adaptive estimator (33). Genes were considered differentially expressed if |log_2_FC| ≥ 0.585 and FDR ≤ 0.1. For heatmap and clustering, the normalized expression matrix was transformed by way of the shifted logarithm (base 2). Next, gene expression was standardized to mean zero and unit standard deviation while the hierarchical clustering of genes and the heatmap were generated by the R package pheatmap v.1.0.12 with the Pearson correlation coefficient as the distance metric (34). Gene set variation analysis (GSVA) was used to summarize the multiple gene expressions of a given pathway into a sample-wise representative score. Genes were retrieved from Gene Ontology’s “type I interferon signaling” (GO: 0060337), “type I interferon production” (GO:0032606), and Reactome’s “interferon alpha/beta signaling” (R-HSA-909733.5) pathways. Patient information is described in Supplementary Table 1. The statistical significance of GSVA scores was tested separately for within- and between-patient comparisons. Within patient comparisons, the significance between scores was tested *via* a non-parametric permutation procedure using the R package exactRankTests v.0.8-34. Independent samples were tested by the exact Independence test implemented in the coin v.1.4-2 package. All *p*-values reported are two-tailed and nominal.

### Real Time RT-qPCR

Total RNA from whole blood cells was isolated by way of the PAXgene Blood RNA kit (Qiagen) according to the manufacturer’s recommendations. Total RNA was obtained from skin samples, cDNA was synthesized, and real-time polymerase chain reactions (quantitative RT-PCR) were performed as previously described (35). Oligonucleotide sequences used in the RT–qPCR assays are displayed in Supplementary Table 6. The reactions were incubated in the StepOnePlus^®^ real-time PCR equipment (Applied Biosystems, United States) as described (36). For each sample, the cycle- threshold (CT) means of the genes of interest (*IFNB*, *IFNAR1*, *IFI16*, *TBK1*, *EIF2AK2*, and *MX1*) were normalized by the CT mean of the reference gene *RPL13a* (ThermoFisher Scientific, United States). The relative gene expression analysis was performed utilizing the 2^–ΔCT^ method for each target gene (37).

### Immunohistochemical and Immunofluorescence Stainings

Histology of skin tissue from NR and ENL patients was carried out as reported previously (38, 39). Standard staining with hematoxylin and eosin (H&E) was done in sections obtained from paraffin-embedded tissue for morphological analysis. For immunohistochemical procedures, tissue sections taken from frozen samples were incubated overnight in 1% normal goat serum (NGS, Sigma-Aldrich, United States) at 4°C with primary anti-IFI16 human monoclonal antibodies (sc-8023, 1G7 clone, Santa Cruz Biotechnology, United States) or anti-CD123 (306002, Biolegend, United States), both at 1:50 dilution, and polyclonal anti-IRF3 (sc-9082, FL-425, Santa Cruz Biotechnology) at 1:100 dilution. An AEC (3-amino-9-ethylcarbazole) solution (Vector Laboratories, United States) was used to detect the primary antibodies (as the manufacturer’s instructions indicate) and monitored under a microscope for a maximum of 20 min to avoid overstaining. The sections were then counterstained with Mayer’s Hematoxylin and mounted with aqueous Mounting Medium (Cell Marque, United States). The primary antibodies were omitted in the negative control slides. Samples were analyzed on a Nikon Eclipse E400 brightfield optical microscope (Nikon Instruments Inc., United States); and a minimum of ten random images per sample were evaluated. For immunofluorescence analysis, sections were incubated overnight with primary mouse monoclonal antibodies against IFN-α (21100, MMHA-2 clone, PBL Assay Science, United States), at 1:100 dilution at 4°C, followed by incubation with secondary goat anti-mouse conjugated with Alexa Fluor 594^®^ (A-11005, Invitrogen, United States) at 1:500 dilution for 1.5 h at room temperature. The nuclei were evidenced by staining with 4’-6’-diamidino-2-phenylindole (DAPI; Molecular Probes, United States) while the slides were mounted with Prolong Gold Antifade (Invitrogen) and analyzed *via* an Axio Observer.Z1 fluorescence microscope equipped with the Colibri.2 illumination system (Carl Zeiss, Germany).

### Western Blot

Biopsied skin lesions were processed for protein extraction in accordance with TRIzol^®^ Reagent protocol (Thermo Fisher Scientific). Proteins isolated from the phenol-ethanol supernatant were dialyzed against 0.1% sodium dodecyl sulfate (SDS, Sigma-Aldrich) as previously described (40). The amount of protein in the samples was measured by the Bradford method with the Pierce Coomassie reagent (Thermo Fisher Scientific). Total protein was separated by 12% SDS-polyacrylamide gel electrophoresis (SDS-PAGE), transferred to the nitrocellulose membrane (G.E. Healthcare Life Sciences, United States), and incubated overnight at 4°C with rabbit polyclonal anti-MX1 antibody (13750-1-AP, Proteintech, United States; 1:1,000 dilution), followed by HRP-conjugated anti-rabbit IgG (A16104, Thermo Fisher Scientific; 1:5,000 dilution). Loading control was assessed *via* GAPDH quantification. Membranes were incubated for 2 h with mouse monoclonal anti-GAPDH antibody (sc-32233, 6C5 clone, Santa Cruz Biotechnology; 1:1,000 dilution) followed by HRP-conjugated, anti-mouse IgG (A16072, Thermo Fisher Scientific; 1:10,000 dilution). Protein bands were detected by chemiluminescence using the Amersham ECL Western Blotting Kit (G.E. Healthcare Life Sciences). Relative protein levels were analyzed *via* ImageJ software.

### Flow Cytometry Analysis

Peripheral blood mononuclear cells (PBMCs) were isolated from whole blood by gradient centrifugation using Ficoll-Paque (G.E. Healthcare Life Sciences). Purified cells were suspended in PBS containing 0.5 M ethylenediamine tetraacetic acid and 10% BSA treated with Fc receptor blocking (human TruStainFcX; Biolegend) and then labeled with the extracellular antibody cocktail for 30 min at 4°C. The following fluorochrome-conjugated human antibodies were used for identification of circulating pDCs: Lineage-FITC (αCD19: 302206, HIB19 clone, Biolegend; αCD20: 302304, 2H7 clone, Biolegend; αCD56: 304606, MEM-188 clone, Biolegend; αCD3: 300306, HIT3a clone, Biolegend; αCD14: 347493, MφP9 clone, BD Bioscience, United States and αCD16: 555406, 3G8 clone, BD Bioscience), αCD123-PerCP/Cy5.5 (306016, 6H6 clone, Biolegend), αCD11c-APC/Cy7 (3372118, Bu15 clone, Biolegend), and αBDCA4-APC (354506, 12C2 clone, Biolegend). After extracellular labeling, the cell suspension was fixed with a 2% paraformaldehyde solution. For experiments in which intracellular staining was performed, the pDCs were identified similarly to what has been described above, except for the exclusion of antibodies that make up the lineage cocktail and the replacement of αBDCA4-APC by αBDCA2-BV421 (354212, 201A clone, BD Bioscience).

Next, fixed cells were permeabilized with 2% saponin and incubated with intracellular antibodies against TLR9 (ab134368, 26C593.2 clone, Abcam, United States) and TLR7 (ab28048, 4F4 clone, Abcam) conjugated with Alexa Fluor^®^ 488 and Alexa Fluor^®^ 647, respectively. Cells were assessed using BD FACSAria™ flow cytometer (BD Bioscience); and the resulting data were analyzed by FlowJo V10 software (BD Bioscience, United States).

### *In vitro* Stimulation Assays

2 × 10^6^ PBMCs from healthy individuals were stimulated with 0.5 μM CpG-A (ODN-2216, InvivoGen, United States) or 20 μg/mL of *M. leprae* whole cell sonicate (NR-19329, BEI Resources, United States). In parallel, 50 μg/mL of thalidomide (ab120032, Abcam) were added or not to the cultures, which were kept at 37°C in a 5% CO_2_ atmosphere for 24 h. Supernatants were harvested and stored at −20°C until TNF and IFN-I measurements.

### Biological Assay of Type I IFN

Type I interferon levels were quantified using the Hek-Blue™ IFNα/β (hkb-ifnab, InvivoGen) biological assay. Twenty microliters per well of conditioned supernatants from stimulated PBMCs or synthetic IFNα/β (Biosintetica, Brazil), utilized to build the standard curve, were added to a 96-well cell culture plate (Corning, United States). A volume of 180 μL from a cell suspension containing 2.8 × 10^5^ Hek-Blue cells/mL was added to each well; and the cultures were maintained at 37°C in a CO_2_ atmosphere for 24 h. Afterward, 20 μL of the supernatants were transferred to another plate and 180 μL of QUANTI-Blue™ (InvivoGen) were added to the wells. The plates were incubated at 37°C for 15 min in the dark. The optical density was determined at 620 nm with the BioTek Eon™ Microplate Spectrophotometer (BioTek, United States).

### Enzyme-Linked Immunosorbent Assay

IFN-β detection was performed in serum samples using the immunoenzymatic sandwich VeriKine Human IFN Beta ELISA kit (41410, PBL assay science), according to the manufacturer’s protocol. TNF levels in conditioned supernatants from stimulated PBMCs were measured by the Human DuoSet kit (DY210, R&D Systems, United States) as recommended. The reading of optical densities was measured *via* the SpectraMax^®^ 190 Absorbance Microplate Reader (Molecular Devices, United States); and the data were analyzed by SoftMax^®^ Pro Software version 5.3.

### Statistical Analysis

Comparisons between two groups were assessed by the two-tailed Student *t* test for normally distributed data. Comparisons between more than two groups with normally distributed data were addressed *via* variance analysis (ANOVA) using Bonferroni’s correction for multiple testing. Alternatively, the non-parametric Mann Whitney test was chosen to analyze unpaired data and the Wilcoxon test, for paired data. Non-normal comparisons among more than two groups were performed *via* the Kruskal–Wallis test with Dunn’s multiple comparison *post hoc* test. Statistical analysis was done *via* GraphPad Prism version 9.0.0 (GraphPad Software, United States); and the adopted statistical significance level was *p* < 0.05.

## Results

### Type I IFN Signaling Is Upregulated in the Whole Blood of Erythema Nodosum Leprosum Patients

**FIGURE 1:**
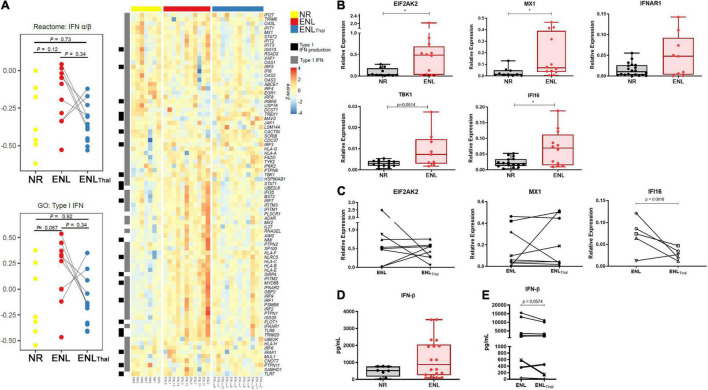
Type I IFN pathway is positively regulated in the blood of ENL patients. Whole blood cells of non-reactional multibacillary leprosy patients (NR – *n* = 7, *yellow*), ENL patients (ENL – *n* = 11, *red*), and thalidomide-treated ENL patients (ENL_Thal_ – *n* = 12, *blue*) were properly collected and RNA was extracted using the PAXgene protocol. RNAseq was performed at NextSeq 550 platform and data analysis was performed using Salmon pipeline, followed by DESeq2 analysis to determine differentially- expressed genes. **(A)** *On the left*: Pathway score for the IFN-I pathway was determined using reactome IFN α/β and gene ontology type I IFN databases. *On the right*: Heatmap including type I IFN genes (*gray*) and Type I IFN production (*black*) from gene ontology database. **(B,C)** RNA from whole blood cells was extracted from NR, untreated ENL, or ENL patients on day 7 of thalidomide therapy (ENL_Thal_) to validate the gene expression of ISG genes by RT-qPCR. *RPL13* was used as an endogenous control. **(B)** Gene expression levels were determined in NR (*black*) and ENL patients (*red*) for *EIF2AK2* (NR = 14, ENL = 14); *MX1* (NR = 14, ENL = 15); *IFNAR1* (NR = 20, ENL = 9); *TBK1* (NR = 13, ENL = 9), and *IFI16* (NR = 16, ENL = 12). *p < 0.05. **(C)** Follow-up gene expression analysis of *EIF2AK2* (*n* = 8), *MX1* (*n* = 9), and *IFI16* (*n* = 5) in ENL patients before (ENL) and at day 7 of thalidomide treatment (ENL_Thal_). **(D,E)** IFN-β levels in serum samples of NR and ENL leprosy patients. **(D)** Serum concentrations of IFN-β were evaluated by enzyme-linked immunosorbent assay (ELISA) in NR (*n* = 8, *black*) and ENL patients (*n* = 18, *red*). **(E)** Serum concentrations of IFN-β in a longitudinal follow-up measurement of leprosy patients diagnosed with ENL and after 7 days of thalidomide treatment (*n* = 13). Box plots show median, interquartile range, sample minimum and maximum. Each dot represents a donor.

Since ENL is frequently accompanied by systemic symptoms, an assessment of the IFN-I pathway in the blood compartment was performed. RNAseq data comparing the whole blood of ENL and NR patients revealed an increased enrichment of type I IFN genes in the former patients when compared to the latter, in whom, respectively, the reactome IFN-α/β pathway (median difference = 0.3, *p* = 0.12) presented a median pathway score of −0.09 and −0.39. Using the gene ontology Type I IFN annotation, the same comparison resulted in median scores of 0.32 and −0.27, respectively, for ENL and NR (median difference = 0.59, *p* = 0.067; Figure 1A). The *IFITM1* (a 1.85-fold increase), *GBP2* (a 1.6-fold increase), and *IFITM2* genes (a 1.62-fold increase) were the top three upregulated IFN I-related genes found in the ENL whole blood samples (Figure 1A). Interestingly, the genes associated with IFN I production such as *TLR8* and *TLR9* were also positively regulated in whole blood cells of ENL patients (Supplementary Table 7). Moreover, the analysis of paired samples from ENL patients before treatment of reaction (ENL) and on day 7 of thalidomide administration (ENL_Thal_) points to an overall decreasing trend in the IFN-I pathway scores for both datasets during treatment (Figure 1A). The expression of three type I IFN-regulated genes (*EIF2AK2, MX1*, and *IFI16*) and two IFN-I pathway genes (*TBK1* and *IFNAR1*) was further explored by RT-qPCR analysis (Figure 1B). The ISGs were selected based on their upregulation in other diseases with a type I IFN signature in the blood (41–44). Despite the observation of heterogeneous behavior among ENL patients, significantly higher mRNA levels of *EIF2AK2, MX1, IFI16*, and *TBK1* were detected in ENL when compared to those of NR patients (with respective *p* values of 0.0387; 0.0358; 0.0331; and 0.0514). The RT-qPCR analysis of the *EIF2AK2* and *MX1* genes in paired samples of ENL and ENL_Thal_ did not show a clear decrease in the expression of these genes in blood cells after 7 days of thalidomide treatment (Figure 1C). *EIF2AK2* mRNA levels were reduced in four patients after treatment while no change was observed in the remaining three. Similar behavior was observed regarding the *MX1* gene (Figure 1C). Clinical data of the patients enrolled in the experiments shown in Figures 1A–C can be seen in Supplementary Table 1.

IFN-β concentration (expressed as pg/mL) was then quantified in the serum samples of NR (*n* = 8) and ENL patients (*n* = 18). Although no statistically significant differences were observed, the median IFN-β serum levels were roughly 1.7-fold higher in ENL than in NR patients [876.8 (IQR: 251.9–2058) versus 521.5 (IQR: 169.1–765.4), respectively, *p* = 0.1961] (Figure 1D). To verify whether the IFN-β serum levels observed in ENL patients are affected by thalidomide, a longitudinal follow-up of these patients was performed during treatment. A decrease in IFN-β serum levels was observed in 9 out of the total 13 patients at day 7 of thalidomide treatment (Figure 1E; *p* = 0.0574). Clinical data of patients enrolled in the assays in Figures 1D,E are shown in Supplementary Table 2. In summary, these results suggest the upregulation of the IFN-I signature in the peripheral blood of ENL patients and the tendency to decrease this upregulation during thalidomide treatment.

### Type I IFN Pathway Genes Are Upregulated in Erythema Nodosum Leprosum Skin Lesions

**FIGURE 2:**
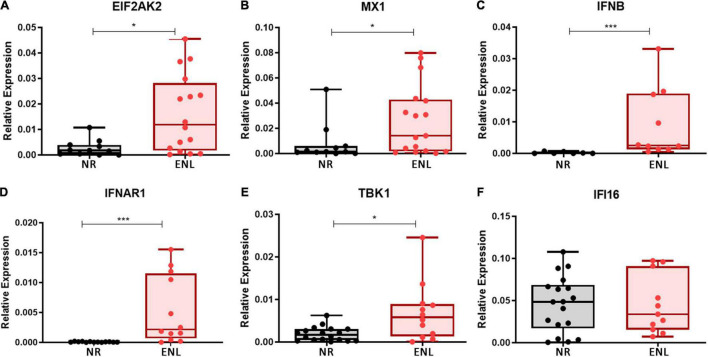
Type I IFN signature genes are upregulated in ENL skin lesions. Skin lesions of non-reactional multibacillary patients (NR, *black*) or ENL patients (ENL, *red*) were processed in TRIzol^®^ reagent for RNA extraction and gene expression was determined by RT-qPCR for the following ISGs: **(A)** *EIF2AK2* (NR = 12, ENL = 16); **(B)** *MX1* (NR = 11, ENL = 17); **(C)** *IFNB* (NR = 7, ENL = 10); **(D)** *IFNAR1* (NR = 13, ENL = 12); **(E)** *TBK1* (NR = 18, ENL = 12); and **(F)** *IFI16* (NR = 18, ENL = 11). *RPL13* was used as an endogenous control. Box plots show median, interquartile range, sample minimum and maximum. Each dot represents a donor. *p < 0.05; ***p < 0.001.

To investigate whether type I interferons play a role in ENL immunopathogenesis on the reaction site, the mRNA levels of the IFN-I-related genes previously evaluated in the blood were then analyzed in ENL skin lesions by RT-qPCR. For comparison, cutaneous lesions of NR patients, previously described as already exhibiting an IFN-β signature (45), were also studied and the mRNA levels of the *IFNB* gene, monitored. Clinical data of patients enrolled in this analysis are shown in Supplementary Table 3. In contrast to NR, the majority of ENL skin lesions showed detectable levels of all genes investigated. Although a heterogeneous expression profile was observed among patients, significantly higher levels of mRNA for the *EIF2AK2* (*p* = 0.0131), *MX1* (*p* = 0.0469), *IFNB* (*p* = 0.0004), *IFNAR1* (*p* = 0.0001), and *TBK1* (*p* = 0.0150) genes were detected in ENL versus NR lesions (Figures 2A–E). The expression of the *IFI16* gene was also noted, presenting similar levels in both groups of patients (*p* = 0.9824; Figure 2F). These initial data suggest that the type I IFN pathway is activated in ENL skin lesions in that IFN-I-associated genes showed higher expression levels than those found in the NR lesions.

### The IFN-I Pathway Is Downregulated During Thalidomide Treatment in Erythema Nodosum Leprosum Skin Lesions

Since thalidomide is known to drastically ameliorate ENL cutaneous and systemic symptoms after a brief period of treatment (24, 25) the next step involved investigating the effect of this drug on the expression of type I IFN-associated genes/molecules in ENL lesions of leprosy patients during thalidomide therapy. For this purpose, at day 7 of thalidomide treatment, ENL skin biopsies were collected for analysis (ENL_Thal_ group) and compared with the pre-treatment lesions. Clinical data of the patients enrolled in this section of the study are shown in Supplementary Table 4.

**FIGURE 3:**
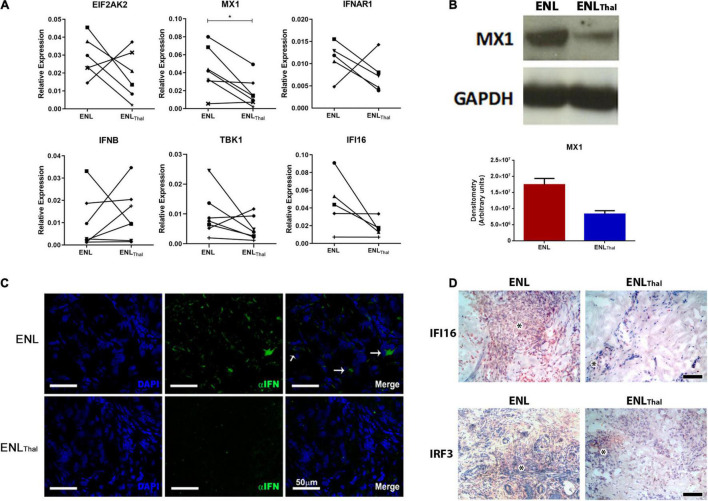
Thalidomide treatment decreases IFN-I pathway activity in skin lesions of ENL patients. **(A)** Follow-up of ENL patients before (ENL) and after 7 days of thalidomide treatment (ENL_Thal_). Skin lesions were processed in TRIzol^®^ for RNA extraction. Gene expression was determined by RT-qPCR for the following genes: *EIF2AK2* (*n* = 6), *MX1* (*n* = 7), *IFNAR1* (*n* = 5), *IFNB* (*n* = 7), *TBK1* (*n* = 7), and *IFI16* (*n* = 7). *RPL13* was used as an endogenous control. **(B)** *Top*– Longitudinal follow-up of MX1 protein levels by Western blot in skin lesions before (ENL = 3) and on Day 7 of thalidomide treatment (ENL_Thal_ = 3). GAPDH was used as an endogenous constitutive gene. *Bottom* – Densitometry analysis displayed in arbitrary units. **(C)** Immunofluorescence for IFN-α (*green*) of skin lesions of ENL patients (*n* = 3) and ENL_Thal_ (*n* = 3). Cell nuclei are in *blue* by DAPI staining. *White* arrows (→) indicate IFN-α labeling in the merge images. Scale bar = 50 μm. **(D)** Representative immunohistochemical analysis of IFI16 and IRF3 proteins in skin lesions of ENL and ENL_Thal_ patients. Inflammatory infiltrate is indicated by the asterisk symbol. The photomicrographs are representative of four patients from each group. Scale bar = 100 μm. *p < 0.05.

Firstly, the transcriptional expression levels of key genes of the IFN-I pathway were analyzed in a follow-up analysis of ENL skin lesion specimens collected from patients before and after initiating thalidomide therapy. Except for *IFNB* mRNA, which displayed a highly heterogeneous patient response, our data showed a decrease in the mRNA levels of *MX1*, *IFNAR1*, *TBK1*, *EIF2AK*, and *IFI16* in most patients under thalidomide treatment. Yet, only *MX1* (*p* = 0.0127; Figure 3A) attained a statistically significant difference.

Next, a longitudinal follow-up analysis of the levels of the MX1 protein by Western blot was performed in ENL and ENL_Thal_ skin lesions. A reduction of about 50% in MX1 protein expression in ENL_Thal_ was detected *via* densitometric analysis (Figure 3B). In addition, IFN-α expression was assessed by immunofluorescence staining of the ENL (*n* = 3) and ENL_Thal_ (*n* = 3) cryosections of skin lesions. In the dermis of ENL lesions, IFN-α (*green*) expression was found in the inflammatory infiltrate. But, after 7 days of treatment, only a faint signal could be detected (Figure 3C). IFI16 protein levels in ENL (*n* = 4) and ENL_Thal_ (*n* = 4) tissues were then monitored by immunohistochemistry. To further complement the study, the protein levels of IRF3, a transcriptional factor that regulates the expression of IFN-I genes and a number of ISGs (46), were also analyzed by immunohistochemistry. The *IRF3* gene was one of the upregulated IFN-I-related genes in the RNAseq analysis of ENL peripheral blood (Figure 1A). IFI16^+^ and IRF3^+^ cells were observed among the inflammatory infiltrates in the ENL dermis (Figure 3D). However, a substantial decrease in IFI16 and IRF3 labeling was seen after 7 days of thalidomide intake (Figure 3D). Altogether, these data suggest that, in ENL skin lesions, thalidomide treatment leads to the downregulation of the IFN-I pathway.

### Thalidomide Inhibits CpG- and *Mycobacterium leprae*-Induced *in vitro* IFN-I Production

**FIGURE 4:**
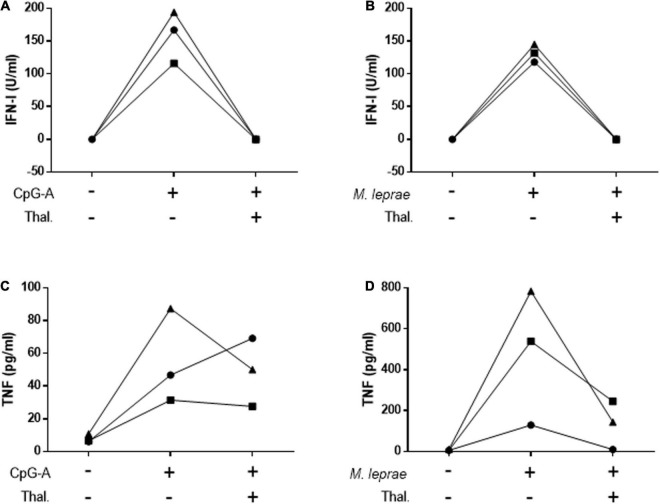
Thalidomide blocks CpG- and *M. leprae*-induced IFN-I secretion by PBMCs. Quantification of IFN-I **(A,B)** and TNF **(C,D)** levels in culture supernatants of PBMCs from three healthy donors stimulated with 0.5 μM CpG-A **(A,C)** or 20 μg/mL *M. leprae* sonicate **(B,D)** and treated or not with 50 μg/mL thalidomide for 24 h. Each individual is represented by a different symbol.

To confirm the capacity of thalidomide to inhibit the IFN-I pathway, PBMCs isolated from HD were stimulated for 24 h with CpG-A 2216, a TLR9 agonist, or *M. leprae* whole cell sonicate in the presence or not of thalidomide. IFN-I was measured in the culture supernatants. TNF quantitation was included as a positive control based on the well-established capacity of thalidomide to block *M. leprae*-induced TNF secretion by PBMCs (47). Figure 4 shows that thalidomide was able to completely block the secretion of IFN-I by the PBMCs of all donors in response to both stimuli (Figures 4A,B). Thalidomide inhibited the production of TNF stimulated by CpG (Figure 4C), and as expected, in *M. leprae*-stimulated cells as well (Figure 4D).

### Involvement of Plasmacytoid Dendritic Cells in Erythema Nodosum Leprosum

**FIGURE 5:**
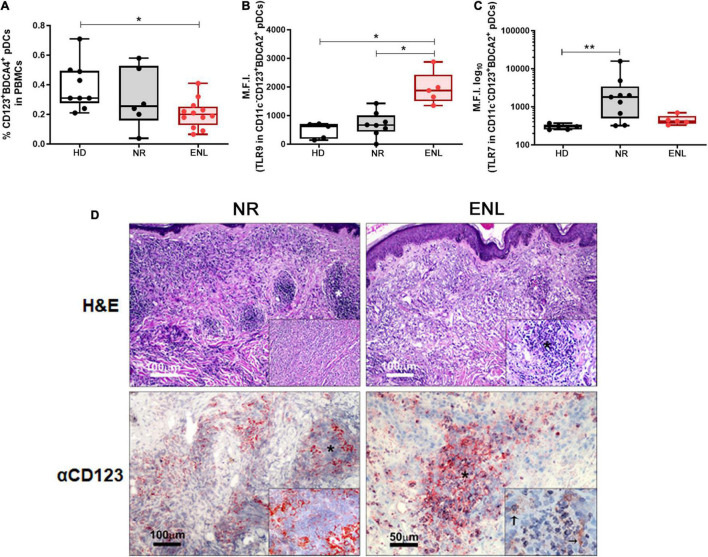
Involvement of plasmacytoid dendritic cells in ENL. **(A–C)** pDC analysis by flow cytometry. **(A)** Frequency of Lin^–^CD11c^–^CD123^+^BDCA4^+^ pDCs among PBMCs isolated from HD (*n* = 9), NR (*n* = 6), and ENL patients (*n* = 12). TLR-9 **(B)** and TLR-7 **(C)** expression levels in pDCs (CD11c^–^CD123^+^BDCA2^+^) present in PBMCs isolated from HD (*n* = 5), NR (*n* = 8), and ENL patients (*n* = 5). Box plots show median, interquartile range, and sample minimum and maximum. Each dot represents a donor. *p < 0.05, **p < 0.01. **(D**, *Top*) H&E staining of skin lesions from NR and ENL patients. Asterisks indicate inflammatory infiltrates in the dermis. Insets show dermal inflammatory infiltrate with macrophages and rare polymorphonuclear cells (NR) and a microabscess with a high concentration of polymorphonuclear cells (ENL). **(D**, *Bottom*) CD123 immunohistochemical expression in skin lesions of NR and ENL patients. CD123 expression is mainly observed in the peripheral regions of the dermal inflammatory infiltrates (NR), while microabscesses in ENL lesions show a marked expression of CD123. Inset shows expression of CD123 close to neutrophils (*arrows*). Images are representative of three NR and three ENL patients.

Plasmacytoid dendritic cells are able to produce large amounts of IFN-I and, when activated, migrate from the blood to the affected tissues (12). Indeed, the cutaneous infiltration of these cells is a histological hallmark in inflammatory and autoimmune diseases affecting the skin, upon which they imprint an IFN-I signature (15–18). In humans, pDCs express a rather specific set of cell surface markers such as the blood dendritic cell antigen 2 (BDCA-2) and BDCA-4, along with a high expression of CD123, the alpha subunit of the IL-3 receptor (10). Therefore, to investigate whether this particular cell type contributes to the IFN-I signature found in ENL lesions, the frequency of pDCs in the blood of NR and ENL patients in comparison to that of HDs was evaluated. Clinical data of all patients enrolled in these subsequent experiments are shown in Supplementary Table 5. Supplementary Figure 1 shows the flow cytometry gating strategy used to identify circulating pDCs (Lin^–^CD11c^–^CD123^+^BDCA4^+^) among PBMCs and the representative plots of the cell population for each study group. *Ex vivo* flow cytometry analysis showed significantly lower frequencies of pDCs in ENL patients when compared to the healthy donor rates (HD = 0.31%, NR = 0.25%, ENL = 0.20%; HD versus ENL, *p* = 0.018; Figure 5A).

Since pDCs are known to produce IFN-I mainly through extracellular nucleic acid recognition *via* TLR7 and TLR9 endosomal receptors (5, 9, 10), the next step aimed to analyze the status of their expression in circulating pDCs. In this *ex vivo* assay, a similar flow cytometry gating strategy was undertaken to identify circulating pDCs, except for the replacement of the anti-BDCA4 by an antibody that recognizes BDCA2 (Supplementary Figure 2). TLR9 expression levels in circulating pDCs (CD11c^–^CD123^+^BDCA2^+^), obtained by mean fluorescence intensity (MFI) values, were about three-fold higher in ENL pDCs when compared to the pDCs of the NR patients and healthy volunteers (HD = 621, NR = 666.5, ENL = 1,875; NR versus ENL, *p* = 0.024; HD versus ENL, *p* = 0.011). TLR9 MFI values of NR patient pDCs did not differ from those found among the healthy volunteers (Figure 5B). In contrast, TLR7 expression presented a significant increase in NR pDCs in comparison to the HD while similar MFI values were observed between the ENL pDCs and their healthy counterparts (HD = 302, NR = 1,803, ENL = 416; HD versus NR, *p* = 0.004; Figure 5C). Interestingly, the MFI median values of TLR7 in NR pDCs were overall around four-fold higher relative to those of ENL patients and HD (Figure 5C).

The presence of CD123^+^ cells in the skin lesions of NR and ENL patients was analyzed. Skin biopsy specimens stained with H&E confirmed the characteristic inflammatory infiltrate observed in NR lesions, namely, a diffuse dermal inflammatory infiltrate with macrophages and rare polymorphonuclear cells. At the same time, microabscesses with a high concentration of polymorphonuclear cells were observed in ENL lesions (Figure 5D, Top). In NR lesions, CD123^+^ cells were primarily found in the peripheral regions of the dermal inflammatory infiltrates. Conversely, in ENL lesions, marked CD123 staining was more in evidence in microabscesses close to neutrophilic infiltration (Figure 5D, Bottom). Altogether, the data generated in this section of the study infer that, in ENL, pDCs may contribute to the production of type I IFNs.

## Discussion

The physiopathological mechanisms involved in ENL, a major complication of leprosy, remain elusive. ENL shares similar features with several of the chronic inflammatory and autoimmune diseases that typically intercalate periods of inflammatory activity with periods of remission. In several of these pathologies, type I IFNs have been shown to play a central role as adjuvants by exacerbating inflammation through the activation of both innate and acquired immune pathways (4, 5). Moreover, pDCs activation *via* nucleic-acid recognition by endocytic TLR7/TLR9 has been implicated as an important source of IFN-I in these diseases (5, 9, 10). In the present study, the production of IFN-I during reaction was investigated based on our previous report suggesting that DNA sensing *via* TLR9 constitutes a major innate immunity pathway involved in the pathogenesis and progression toward ENL. The generated data showed higher activation of the type I IFN pathway both in the skin lesions and blood of ENL patients compared to the corresponding data found in NR patients. Upon treatment with thalidomide, the type-I IFN pathway seems to recede in both compartments. Furthermore, *in vitro* assays have confirmed the capacity of thalidomide to block IFN-I production by PBMCs in response to *M. leprae*. Finally, the decreased frequencies observed in peripheral pDCs, together with their higher TLR9 expression and the presence of CD123 + cells in the ENL skin lesions, imply the involvement of these cells as IFN-I producers during this type of reactional episode.

Although a positive regulation of the type I IFN pathway was detected during ENL, quite heterogeneous behavior was observed among patients with respect to mRNA levels of target genes, the RNAseq profile, and circulating IFN-β levels. A possible reason for this heterogeneity might be traced to the differences each patient encounters regarding the time period covered from the onset of ENL symptoms up to diagnosis and, likewise, in the severity of the reactional episode itself. Another factor that may affect patient response is the time span of MDT treatment since its length may or may not strongly influence the immune status of the patient in any direction (48).

Albeit not a major upregulated pathway in the RNAseq, the observed enrichment in type I Interferon genes in ENL versus NR whole blood correlates with the upregulated IFN I pathway found in whole blood transcriptomics analysis during reversal reaction, the other type of leprosy reaction (49, 50), suggesting that this upregulation could be a common dissonant trait during acute inflammatory reactional episodes. It is noteworthy that IFI16 expression was upregulated in whole blood cells while no difference was observed in ENL skin lesions. Higher anti-IFI16 antibody levels have already been associated with other inflammatory diseases such as SLE (51), rheumatoid arthritis (52), and psoriatic arthritis (53). Besides identifying increased IFI16 gene expression levels in SLE, increased levels have also been reported to be closely related to disease activity (54), pointing to IFI16 as a candidate blood biomarker during an ongoing ENL episode.

Type I interferons play a complex, highly contextually dependent role in infectious diseases, leading to either beneficial or detrimental outcomes for the host. Even during viral infections, in which interferons classically contribute to protection, IFN-I may act by suppressing the immune response, thus favoring a chronic perpetuation of the infection. IFN-I may also be responsible for immunopathology and host morbidity and/or mortality (3). In the case of bacterial pathogens, IFN-I signaling likewise displays diverse effects on host susceptibility; and the mechanisms responsible for these effects are wide and varied (2, 3). In a previous study, the predominance of an IFN-I signature was demonstrated in cutaneous lesions of NR patients (BL/LL) in contrast to the IFN-γ program observed in the self-limiting paucibacillary [tuberculoid (TT) and borderline tuberculoid (BT)] clinical forms of the disease. The authors showed that local IFN-I production, particularly IFN-β produced by infected macrophages, is capable of blocking the anti-microbicidal program induced by IFN-γ, allowing for *M. leprae* persistence and multiplication (45). Complementing these data, in a recent report, the present authors demonstrated that *M. leprae* induced IFN-I in infected macrophages and Schwann cells *via* the cyclic cGMP-cAMP synthase (cGAS)-stimulator of the interferon genes (STING) cytoplasmic nucleic acid sensor pathway. Moreover, silencing the 2′-5′ oligoadenylate synthetase-like gene, the ISG that revealed the highest upregulation, decreased the intracellular viability of *M. leprae* together with a concomitant increase in microbicidal mechanisms (36). Thus, the capacity of *M. leprae* to induce an IFN-I program during the early phases of infection has been considered an important event for successful tissue colonization, constituting a crucial mechanism of bacterial pathogenesis (55). A similar detrimental role for the host of IFN-α/β during tuberculosis (TB) has been supported by studies performed on TB patients and in mouse models of infection (3).

To our knowledge, this is the first time that IFN-I pathway activation has been associated with ENL. Type I IFNs affect a broad spectrum of events implicated in the pathogenesis of several inflammatory and autoimmune diseases acting as adjuvants in exacerbating inflammation and tissue damage (5, 12, 13). Thus, it is tempting to speculate that type I IFNs may play a role in ENL pathogenesis through similar mechanisms. Indeed, the capacity of IFN-I to promote DC maturation/differentiation and increase Th1 differentiation while suppressing Tregs may be linked to the presence of a higher percentage of activated CD4^+^ and CD8^+^ T cells (56), a lower percentage of CD4^+^regulatory T-cells (57), and the emergence of an IFN-γ signature in PBMCs of ENL compared to what has been found among non-reactional LL patients (58). Moreover, it has also been shown that IFN-I enhances the formation of neutrophil DNA extracellular traps (NETs; 59, 60), which is in line with our previous findings showing an increase in spontaneous NETs formation in ENL peripheral neutrophils (61). Lastly, the recently described higher percentage of activated peripheral memory B-cells in ENL patients (62) may to some extent result in the capability of IFN-α/β to promote B-cell activation and antibody production (5, 12, 13).

As often occurs in a number of inflammatory diseases in which type I IFNs play an immunopathological role, the data in the present study suggest that pDCs are important sources of these cytokines in ENL skin lesions. It has been reported that pDCs frequency in the peripheral blood of SLE patients is reduced upon the simultaneous infiltration of these cells in target tissues such as the skin and renal tissue (63–66). Similarly, a significantly lower peripheral frequency of CD123^+^ BDCA4^+^ cells and the concomitant detection of CD123^+^ cells in the inflammatory infiltrate in dermal ENL lesions suggest the migration of pDCs from the blood to the skin during reaction. However, future research is needed to better characterize the IFN-I producer cells as well as the IFN-I subtypes present in ENL skin lesions.

The presence of pDCs in leprosy lesions has been previously described (38). In this study, all CD123^+^ cells found in the lesions were also shown to express BDCA2 and/or BDCA4, specific markers of these cells. The above authors reported higher concentrations of pDCs in the skin lesions of Type I reactional (RR) patients compared to non-reactional ones, inferring that pDCs may also play a role in this type of reactional episode, probably through the capacity of these cells to enhance both innate and adaptive immune responses *via* numerous mechanisms (18).

Evidence suggesting the involvement of pDCs in the type I signature found in ENL is in accordance with our previous data showing a higher expression of TLR9 in PBMCs and skin lesions besides higher circulating levels of mycobacterial and self-TLR9 ligands in ENL in comparison to the levels associated with NR patients (26, 61). pDCs have the capacity to rapidly produce extraordinary amounts of all type I and type III IFNs, primarily through nucleic acid sensing TLR7/TLR9 receptors. Data point to the central role played by extracellular autologous nucleic acids derived from apoptotic and/or necrotic cells and neutrophils undergoing NETosis as major inducers of IFN-I in pDCs in inflammatory and autoimmune diseases such as SLE (9). As regards ENL, circulating levels of the autologous histone-DNA complex and the mycobacterial histone-like protein (Hlp; likely complexed to bacterial DNA) were observed to be significantly higher, which may function as relevant TLR9 ligands leading to IFN-I production by pDCs during ENL (20). Indeed, after initiating MDT, ENL is most frequently observed in patients with high bacterial loads, coinciding with massive bacterial killing and mycobacterial product release. Thus, the bacterial DNA released from cells after *M. leprae* degradation is likely to act as an ENL trigger.

Furthermore, it has been shown that NETs activate pDCs to produce high levels of IFN-α in a TLR9-dependent manner (67). Additionally, the oxidized mitochondrial DNA present in NETs has been demonstrated to activate pDCs *via* the intracellular nucleic acid sensing cGAS/STING pathway (68). Likewise, recent reports support the idea that neutrophils play a central role in ENL pathogenesis (69). Indeed, in contrast to non-reactional LL/BL skin lesions, neutrophils accumulate inside ENL skin lesions, demonstrating an activated phenotype in the skin and blood (70–73). Of note, our previous study also found that ENL neutrophils underwent increased rates of NETosis (61). Abundant NETs were found in ENL skin lesions and increased spontaneous NETs formation was observed in the peripheral neutrophils of ENL patients. Indeed, neutrophils were found in the microabscesses in the vicinity of CD123^+^ cells in ENL skin lesions. Thus, it is likely that NETs could play a role in the activation of pDCs to locally produce IFN-I during the emergence of ENL episodes. Conversely, it has been found that IFN-I can stimulate neutrophils to undergo NETosis. Indeed, a link between the presence of an IFN-I signature and neutrophil-mediated pathological damage has been established in several inflammatory manifestations (59). As such, it is possible that this positive feedback between pDCs and neutrophils may be occurring in ENL skin lesions to result in exacerbated type-I IFN production.

An intriguing observation was the opposite behavior displayed by the expression of TLR7 and 9 within the circulating pDCs of ENL versus NR patients. While TLR9 was upregulated in ENL pDCs, TLR7 was more highly expressed in NR pDCs. Since endosomal and cytoplasmic DNA- and RNA-sensing receptors recognize both autologous and pathogen-derived nucleic acids, evidence points to complex interactions maintaining a delicate equilibrium among these receptors to avoid a detrimental response to the host, culminating in inflammation and autoimmunity (74). In this context, a negative regulatory effect of TLR7 on TLR9-mediated signaling in addition to the TLR9 mRNA expression itself have been described (75). In light of these data, it is reasonable to speculate that it is TLR9, and not TLR7, the predominantly activated receptor in ENL pDCs to generate IFN-I, a finding in line with our previous data suggesting that DNA sensing *via* TLR9 is an important inflammatory pathway in ENL (26). Moreover, distinct functional outcomes attributed to TLR9 versus TLR7 activation have also been reported. In mouse models of SLE, e.g., a more pathogenic signal is primarily associated with TLR7- over TLR9-elicited responses (76). Future research is needed to evaluate the potential significance of the distinct phenotypes observed in the TLR7/9 expressions within the pDCs of NR and ENL in leprosy immunopathogenesis.

Thalidomide is considered to be the most effective drug in the treatment of ENL symptoms, with the added benefit of doing so quite rapidly. Several mechanisms have been considered as possible causes of its overall effectiveness. In principle, the capacity of the drug to inhibit TNF production is presumed to be the most important benefit (20). Nevertheless, thalidomide and its derivatives have a broad range of immunomodulatory effects that have also been examined in other diseases. Most of their immunomodulatory effects are associated with their capacity to interact with Cereblon (CRBN), a component of the Cul4A-E3 ubiquitin-ligase complex, thus interfering in the ubiquitination process of a variety of targets (77).

Our data suggest a decrease in type I IFN pathway activity in the skin lesions and blood of ENL patients undergoing thalidomide treatment. Significantly, the complete inhibition by thalidomide of CpG-A and *M. leprae*-induced IFN-I production in PBMCs *in vitro* imply that the drug could directly inhibit this cytokine production, an observation in accordance with a study showing that thalidomide and its derivatives inhibit TLR-induced IFN-I production in the THP-1 monocytic cell line (78). These authors showed that treatment with lenalidomide, a thalidomide derivative, disrupted the interaction between the rabex-5 protein and CRBN, which is important for inhibiting IFN-I production in this cell line, indicating that this anti-inflammatory property of rabex-5 should be further explored. However, another study by the same authors demonstrated that the inhibition of IFN-I production promoted by thalidomide and its analogs primarily occurred by affecting the TRIF/IRF3 pathway independently of CRBN in peritoneal macrophages of CRBN knockout mice. Conversely, the CRBN knock down in THP-1 monocytes decreased IFN-I production, suggesting a different behavior based on cell type (79). Nonetheless, these reports unquestionably link the immunomodulatory effects of thalidomide with the type-I IFN pathway. In the present study, thalidomide seemed to primarily affect the production of IFN-I by pDCs since the latter accounts for most of the TLR9-induced IFN-I production in PBMCs. The mechanisms and targets that are responsible for thalidomide action in inhibiting IFN-I production by pDCs deserve further exploration.

The development of a safe and effective alternative to both steroids and thalidomide for ENL treatment is an urgent need. The present study pointedly reveals an exacerbation of the type I IFN pathway in the skin lesions and blood of leprosy patients undergoing ENL. The present results also suggest the involvement of ENL pDCs in type I IFN production, the major source of these cytokines in the body. The pDC/IFN-I pathway has been specifically implicated in the pathogenesis of skin manifestations in chronic inflammatory/autoimmune diseases in which an enrichment of pDCs in the inflammatory infiltrate in association with an IFN-I signature has been demonstrated (9, 18). Of note, a recent therapeutic approach in the treatment of cutaneous lupus targeting pDCs has shown promising results (80), which may prove useful in treating ENL as well. Moreover, the data herein generated open new avenues in the process of identifying new biomarkers for early ENL diagnosis that could pave the way toward the better management of reactional patients.

## Data Availability Statement

The datasets presented in this study can be found in online repositories. The names of the repository/repositories and accession number(s) can be found below: https://www.ncbi.nlm.nih.gov/geo/, GSE198609.

## Ethics Statement

The studies involving human participants were reviewed and approved by FIOCRUZ Committee for Ethics in Research. The patients/participants provided their written informed consent to participate in this study.

## Author Contributions

TRos, MAM, NL, and TRod rationale for the study and manuscript preparation. MOM, RP, VS, LR, and MP designed the study and performed the project supervision. MOM, RP, and MP were responsible for funding acquisition. TRos, MAM, NL, TRod, AD, and FC for performing experiments. AS was responsible for patients’ recruitment. HF was responsible for histopathology. TL-C was responsible RNAseq data analysis and graph generation. TRos, MAM, NL, TRod, AD, MG, and TA analyzed the results. MOM, RP, and VS were responsible for manuscript revision. TRos, MAM, NL, TRod, AD, LR, and MP were responsible for writing the original manuscript draft while MOM, RP, and VS were in charge of revising it. All authors contributed to the article and approved the submitted version.

## Conflict of Interest

The authors declare that the research was conducted in the absence of any commercial or financial relationships that could be construed as a potential conflict of interest.

## Publisher’s Note

All claims expressed in this article are solely those of the authors and do not necessarily represent those of their affiliated organizations, or those of the publisher, the editors and the reviewers. Any product that may be evaluated in this article, or claim that may be made by its manufacturer, is not guaranteed or endorsed by the publisher.

## Footnotes

1
https://www.bioinformatics.babraham.ac.uk/projects/fastqc/

2
http://refgenomes.databio.org/v2/asset/fa159612d40b1bedea9a279eb24999b3d27145f9dd70dcca/salmon_index/splash?tag=default

## Supplementary Material

Supplementary Figure 1 Flow cytometry gating strategy to identify pDCs in PBMCs. Lumps and doublet cells were excluded using the parameters of frontal dispersion measured by area (FSC-A, forward scatter-area) versus frontal dispersion measured by height (FSC-H, forward scatter-height). Next, the PBMC region was selected by FSC-A and lateral dispersion was measured by SSC-A area (side scatter). In the region of PBMCs, pDCs were identified as lineages (CD3, CD14, CD19, CD20, and CD56)^–^, CD11c^–^, CD123^+^, and BDCA4^+^. Representative plots of pDCs for each study group are shown by using CD123 and BDCA4 markers. HD, healthy donor; NR, non-reactional multibacillary patient; and ENL, erythema nodosum leprosum.

Supplementary Figure 2 Gating strategy and representative histograms of TLR9 and TLR7 levels in pDCs. Lumps and doublet cells were excluded using the parameters of frontal dispersion measured by area (FSC-A, forward scatter-area), versus frontal dispersion measured by height (FSC-H, forward scatter-height). The PBMC region was selected by FSC-A and lateral dispersion measured by SSC-A area (side scatter). In the region of PBMCs, pDCs were identified as CD11c^–^, CD123^+^ and BDCA2^+^. The expression levels of TLR9 and TLR7 by pDCs are shown in the representative histograms. HD, healthy donor; NR, non-reactional multibacillary patient, ENL, erythema nodosum leprosum; and FMO, fluorescence minus one.
