# Supplementary material for: The Type I Interferon Pathway Is Upregulated in the Cutaneous Lesions and Blood of Multibacillary Leprosy Patients With Erythema Nodosum Leprosum
Source: Front Med (Lausanne). 2022 Jun 6;9:899998. doi: 10.3389/fmed.2022.899998 (PMC9208291; doi:10.3389/fmed.2022.899998)
Supplement: Supplementary file 1 [file Table_1.DOCX]

**Table S1 - Patients included in RNAseq experiments and RT-qPCR from whole blood samples in figures 1a,b and c.** M- Male. F- Female. NR- Non reactional, LL – Lepromatous leprosy, BL – Borderline Lepromatous, ENL – Erythema Nodosum Leprosum, ENL_Thal_- ENL patient at 7^th^ day of thalidomide treatment. BI – Bacilloscopic Index. AD – At diagnostics; DT – During treatment; AT – After treatment. Y – Yes, N- No

| **Patient ID** | **Sex** | **Age** | **Clinical**  **Form** | **BI** | **Reaction** | **Reaction**  **diagnostics** | **First**  **episode** | **Symbol** | **Type of analysis** |
| --- | --- | --- | --- | --- | --- | --- | --- | --- | --- |
| **NR1** | F | 44 | LL | 5 | - | - | - |  | RNAseq |
| **NR2** | M | 38 | LL | 5 | - | - | - |  | RNAseq & RT-qPCR |
| **NR3** | M | 22 | LL | 4.75 | - | - | - |  | RNAseq & RT-qPCR |
| **NR4** | M | 44 | LL | 5 | - | - | - |  | RNAseq |
| **NR5** | M | 62 | LL | 5.25 | - | - | - |  | RNAseq |
| **NR6** | M | 32 | LL | 4.75 | - | - | - |  | RNAseq & RT-qPCR |
| **NR7** | M | 54 | LL | 6 | - | - | - |  | RNAseq |
| **NR8** | M | 17 | LL | 5.5 | - | - | - |  | RNAseq |
| **NR9** | M | 28 | LL | 5.75 | - | - | - |  | RNAseq |
| **NR10** | F | 64 | BL | 3.25 | - | - | - |  | RT-qPCR |
| **NR11** | M | 53 | BL | 5 | - | - | - |  | RT-qPCR |
| **NR12** | M | 59 | BL | 2.25 | - | - | - |  | RT-qPCR |
| **NR13** | F | 33 | LL | 3 | - | - | - |  | RT-qPCR |
| **NR14** | F | 72 | BL | 1.75 | - | - | - |  | RT-qPCR |
| **NR15** | F | 30 | BL | 4 | - | - | - |  | RT-qPCR |
| **NR16** | M | 47 | LL | 5 | - | - | - |  | RT-qPCR |
| **NR17** | M | 83 | LL | 4.5 | - | - | - |  | RT-qPCR |
| **NR18** | M | 55 | BL | 0.5 | - | - | - |  | RT-qPCR |
| **NR19** | F | 51 | BL | 1 | - | - | - |  | RT-qPCR |
| **NR20** | M | 63 | BL | 3.75 | - | - | - |  | RT-qPCR |
| **NR21** | M | 77 | BL | 3.3 | - | - | - |  | RT-qPCR |
| **NR22** | M | 72 | BL | 0 | - | - | - |  | RT-qPCR |
| **NR23** | F | 67 | LL | 5.7 | - | - | - |  | RT-qPCR |
| **NR24** | F | 24 | LL | 5 | - | - | - |  | RT-qPCR |
| **NR25** | M | 54 | BL | 2.25 | - | - | - |  | RT-qPCR |
| **NR26** | M | 74 | BL | 3.25 | - | - | - |  | RT-qPCR |
| **NR27** | M | 41 | BL | 4.25 | - | - | - |  | RT-qPCR |
| **NR28** | F | 49 | LL | 5 | - | - | - |  | RT-qPCR |
| **NR29** | F | 55 | LL | 5.5 | - | - | - |  | RT-qPCR |
| **NR30** | M | 57 | LL | 4.25 | - | - | - |  | RT-qPCR |
| **NR31** | M | 34 | LL | 5 | - | - | - |  | RT-qPCR |
| **NR32** | M | 28 | BL | 3.5 | - | - | - |  | RT-qPCR |
| **NR33** | M | 46 | LL | 5 | - | - | - |  | RT-qPCR |
| **NR34** | M | 12 | BL | 3.25 | - | - | - |  | RT-qPCR |
| **NR35** | F | 36 | LL | 2 | - | - | - |  | RT-qPCR |
| **ENL1; ENL_Thal_ 1** | M | 40 | LL | 5 | ENL | AT | Y | * | RNAseq & RT-qPCR |
| **ENL2; ENL_Thal_ 2** | M | 34 | LL | 5.5 | ENL | AD | Y |  | RNAseq & RT-qPCR |
| **ENL3** | M | 43 | LL | 5 | ENL | AT | N |  | RNAseq & RT-qPCR |
| **ENL4; ENL_Thal_ 4** | M | 34 | LL | 4.5 | ENL | DT | Y |  | RNAseq |
| **ENL5; ENL_Thal_ 5** | M | 32 | LL | 5.5 | ENL | DT | Y |  | RNAseq |
| **ENL6** | M | 22 | LL | 5 | ENL | AD | N |  | RNAseq |
| **ENL7; ENL_Thal_ 7** | M | 45 | LL | 5.5 | ENL | DT | Y |  | RNAseq |
| **ENL8; ENL_Thal_ 8** | F | 78 | LL | 4.75 | ENL | DT | N |  | RNAseq |
| **ENL9; ENL_Thal_ 9** | M | 39 | LL | 4 | ENL | DT | N |  | RNAseq |
| **ENL10; ENL_Thal_ 10** | F | 36 | LL | 5.5 | ENL | DT | N |  | RNAseq |
| **ENL11; ENL_Thal_ 11** | M | 69 | LL | 5 | ENL | DT | N |  | RNAseq |
| **ENL12; ENL_Thal_ 12** | M | 38 | LL | 5 | ENL | DT | N | ● | RNAseq & RT-qPCR |
| **ENL13** | M | 47 | LL | 5 | ENL | AD | Y |  | RNAseq |
| **ENL14** | F | 76 | LL | 4.5 | ENL | DT | Y |  | RNAseq & RT-qPCR |
| **ENL15** | M | 38 | LL | 5 | ENL | AT | N |  | RNAseq |
| **ENL16; ENL_Thal_ 13** | M | 21 | LL | 3 | ENL | AT | N | ◊ | RT-qPCR |
| **ENL17; ENL_Thal_ 14** | M | 27 | LL | 0 | ENL | AD | Y | 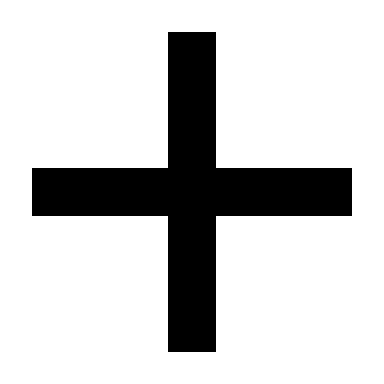 | RT-qPCR |
| **ENL18; ENL_Thal_ 15** | M | 31 | LL | 4.75 | ENL | DT | Y | 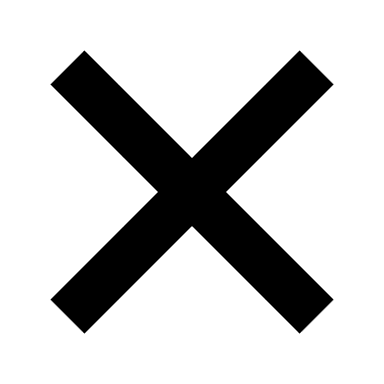 | RT-qPCR |
| **ENL19; ENL_Thal_ 16** | M | 22 | LL | 4.5 | ENL | DT | Y | ▼ | RT-qPCR |
| **ENL20; ENL_Thal_ 17** | M | 27 | LL | 3.25 | ENL | AT | N | ▲ | RT-qPCR |
| **ENL21** | F | 69 | LL | 3.5 | ENL | AT | Y |  | RT-qPCR |
| **ENL22; ENL_Thal_ 18** | M | 52 | LL | 5 | ENL | AT | N | ■ | RT-qPCR |
| **ENL23; ENL_Thal_ 19** | M | 23 | LL | 4.57 | ENL | AT | Y | ♦ | RT-qPCR |
| **ENL24; ENL_Thal_ 20** | M | 26 | BL | 3.75 | ENL | AT | Y | ○ | RT-qPCR |
| **ENL25; ENL_Thal_ 21** | M | 41 | LL | 0 | ENL | AT | N |  | RT-qPCR |
| **ENL26** | M | 44 | LL | 5 | ENL |  |  |  | RT-qPCR |
| **ENL27; ENL_Thal_ 22** | M | 61 | LL | 4 | ENL | DT | Y | □ | RT-qPCR |
| **ENL28** | M | 22 | LL | 5 | ENL | DT | Y |  | RT-qPCR |
| **ENL29** | M | 28 | BL | 3,5 | ENL | AT | Y |  | RT-qPCR |
| **ENL30** | F | 41 | BB | 2 | ENL | AT | N |  | RT-qPCR |
| **ENL31** | M | 50 | BL | 5 | ENL | DT | N |  | RT-qPCR |
| **ENL32** | M | 55 | LL | 5 | ENL | AT | Y |  | RT-qPCR |
| **ENL33** | M | 69 | LL | 5 | ENL | AD | Y |  | RT-qPCR |
| **ENL34; ENL_Thal_ 23** | M | 35 | LL | 4,5 | ENL | DT | Y |  | RT-qPCR |
| **ENL_Thal_ 3** | F | 50 | LL | 5 | ENL | DT | Y |  | RNAseq |
| **ENL_Thal_ 6** | M | 40 | LL | 5 | ENL | AT | Y |  | RNAseq |
