# Supplementary material for: The Type I Interferon Pathway Is Upregulated in the Cutaneous Lesions and Blood of Multibacillary Leprosy Patients With Erythema Nodosum Leprosum
Source: Front Med (Lausanne). 2022 Jun 6;9:899998. doi: 10.3389/fmed.2022.899998 (PMC9208291; doi:10.3389/fmed.2022.899998)
Supplement: Supplementary file 2 [file Table_2.DOCX]

**Table S2 - Patients included in IFNβ measurement in serum samples in figures 1d and 1e .** M- Male. F- Female. NR- Non reactional, LL – Lepromatous leprosy, ENL – Erythema Nodosum Leprosum, ENL_Thal_- ENL patient at 7^th^ day of thalidomide treatment. BI – Bacilloscopic Index. AD – At diagnostics; DT – During treatment; AT – After treatment. Y – Yes, N- No

| **Patient ID** | **Sex** | **Age** | **Clinical**  **Form** | **BI** | **Reaction** | **Reaction**  **diagnostics** | **First**  **episode** | **Symbol** |
| --- | --- | --- | --- | --- | --- | --- | --- | --- |
| NR2 | M | 39 | LL | 5 | - | - | - |  |
| NR36 | M | 50 | LL | 4.5 | - | - | - |  |
| NR37 | M | 37 | LL | 2.75 | - | - | - |  |
| NR38 | M | 26 | LL | 5.25 | - | - | - |  |
| NR39 | M | 33 | LL | 0 | - | - | - |  |
| NR40 | M | 74 | LL | 1 | - | - | - |  |
| NR41 | F | 42 | LL | 0.25 |  | - | - |  |
| ENL16, ENL_Thal_13 | M | 21 | LL | 2.5 | ENL | AT | N | ♦ |
| ENL17, ENL_Thal_14 | M | 27 | LL | 0 | ENL | AD | Y | ▲ |
| ENL18,ENL_Thal_15 | M | 31 | LL | 4.75 | ENL | DT | Y | 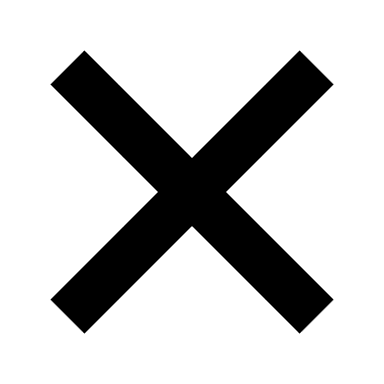 |
| ENL19, ENL_Thal_ 16 | M | 22 | LL | 4.5 | ENL | DT | Y | ● |
| ENL20, ENL_Thal_17 | M | 27 | LL | 3.25 | ENL | AT | Y |  |
| ENL21 | F | 69 | LL | 3.5 | ENL | AT | N |  |
| ENL23, ENL_Thal_ 19 | M | 23 | LL | 4.57 | ENL | AT | Y | ▼ |
| ENL34, ENL_Thal_23 | M | 35 | LL | 4.5 | ENL | DT | Y | 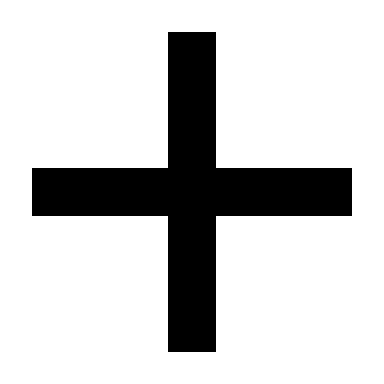 |
| ENL35, ENL_Thal_24 | M | 48 | LL | 4.5 | ENL | AT | Y | * |
| ENL36, ENL_Thal_25 | M | 55 | LL | 2 | ENL | AT | N | □ |
| ENL37, ENL_Thal_26 | M | 62 | LL | 2.6 | ENL | AT | Y | ○ |
| ENL38, ENL_Thal_27 | M | 35 | LL | 4.25 | ENL | DT | Y | 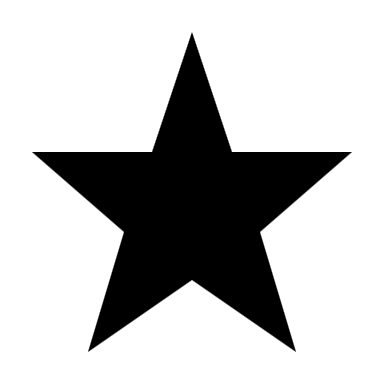 |
| ENL39 | M | 46 | LL | 5 | ENL | AT | Y |  |
| ENL40 | M | 30 | LL | 3.7 | ENL | AD | Y |  |
| ENL41 | M | 39 | LL | 5.5 | ENL | DT | Y |  |
| ENL42, ENL_Thal_28 | M | 51 | LL | 5,25 | ENL | AT | Y | ■ |
| ENL43 | F | 68 | LL | 0 | ENL | AT | Y |  |
| ENL44, ENL_Thal_29 | M | 26 | LL | 3.5 | ENL | DT | N |  |
