# Supplementary material for: The Type I Interferon Pathway Is Upregulated in the Cutaneous Lesions and Blood of Multibacillary Leprosy Patients With Erythema Nodosum Leprosum
Source: Front Med (Lausanne). 2022 Jun 6;9:899998. doi: 10.3389/fmed.2022.899998 (PMC9208291; doi:10.3389/fmed.2022.899998)
Supplement: Supplementary file 3 [file Table_3.DOCX]

**Table S3 - Patients included in RT-qPCR of skin lesions samples in figure 2.** M- Male. F- Female. NR – Non reactional, LL – Lepromatous Leprosy, BL – Borderline Lepromatous, ENL – Erythema Nodosum Leprosum, BI – Bacilloscopic Index. AD – At diagnostics; DT – During treatment; AT – After treatment. Y – Yes, N- No

| **Patient ID** | **Sex** | **Age** | **Clinical Form** | **BI** | **Reaction type** | **Reaction diagnosis** | **First episode** |
| --- | --- | --- | --- | --- | --- | --- | --- |
| NR1 | F | 44 | LL | 5.5 | - | - | - |
| NR2 | M | 39 | LL | 5 | - | - | - |
| NR3 | M | 22 | LL | 5 | - | - | - |
| NR36 | M | 50 | LL | 4.5 | - | - | - |
| NR37 | M | 37 | LL | 2.75 | - | - | - |
| NR42 | M | 17 | LL | 4.75 | - | - | - |
| NR43 | M | 28 | BL | 3.25 | - | - | - |
| NR44 | M | 64 | LL | 5.25 | - | - | - |
| NR45 | M | 69 | BL | 3 | - | - | - |
| NR46 | M | 60 | BL | 0 | - | - | - |
| NR47 | M | 70 | LL | 5.5 | - | - | - |
| NR48 | M | 20 | LL | 5 | - | - | - |
| NR49 | M | 38 | LL | 5 | - | - | - |
| NR50 | F | 49 | LL | 5 | - | - | - |
| NR51 | M | 55 | LL | 5.9 | - | - | - |
| NR52 | M | 46 | LL | 5.85 | - | - | - |
| NR53 | M | 42 | BL | 4.5 | - | - | - |
| NR54 | M | 60 | BL | 4.9 | - | - | - |
| NR55 | M | 23 | LL | 5.95 | - | - | - |
| NR56 | M | 69 | BL | 4.5 | - | - | - |
| NR57 | M | 55 | BL | 2.7 | - | - | - |
| NR58 | F | 40 | LL | 5.95 | - | - | - |
| NR59 | F | 25 | LL | 4 | - | - | - |
| NR60 | M | 55 | LL | 5.85 | - | - | - |
| NR61 | M | 32 | LL | 5.75 | - | - | - |
| NR62 | F | 41 | BL | 4.85 | - | - | - |
| NR63 | M | 19 | LL | 4,85 | - | - | - |
| NR64 | M | 30 | LL | 5 | - | - | - |
| NR65 | F | 69 | LL | 4.9 | - | - | - |
| NR66 | F | 27 | LL | 6 | - | - | - |
| NR67 | M | 41 | LL | 5.7 | - | - | - |
| NR68 | M | 65 | LL | 3.75 | - | - | - |
| ENL1 | M | 40 | LL | 2.75 | ENL | AT | N |
| ENL2 | M | 34 | LL | 5.5 | ENL | DT | Y |
| ENL3 | M | 43 | LL | 3.75 | ENL | AT | N |
| ENL16 | M | 21 | LL | 2.5 | ENL | AT | N |
| ENL17 | M | 27 | LL | 0 | ENL | AD | Y |
| ENL18 | M | 31 | LL | 4.75 | ENL | DT | Y |
| ENL19 | M | 22 | LL | 4.5 | ENL | DT | Y |
| ENL20 | M | 27 | LL | 3.25 | ENL | AT | Y |
| ENL21 | F | 69 | LL | 3.5 | ENL | AT | N |
| ENL23 | M | 23 | LL | 4.57 | ENL | AT | Y |
| ENL35 | M | 48 | LL | 4.5 | ENL | AT | Y |
| ENL36 | M | 55 | LL | 2 | ENL | AT | N |
| ENL37 | M | 62 | LL | 2.6 | ENL | AT | Y |
| ENL38 | M | 35 | LL | 4.25 | ENL | DT | Y |
| ENL45 | M | 31 | BL | 1.25 | ENL | AT | Y |
| ENL46 | M | 70 | LL | 5.57 | ENL | AD | Y |
| ENL47 | F | 50 | LL | 4.5 | ENL | DT | Y |
| ENL48 | M | 64 | LL | 4.5 | ENL | DT | Y |
| ENL49 | M | 42 | LL | 4 | ENL | AD | Y |
| ENL50 | M | 65 | LL | 3.75 | ENL | AT | Y |
| ENL51 | M | 37 | LL | 5 | ENL | AT | N |
| ENL52 | F | 37 | LL | 4.5 | ENL | AT | Y |
| ENL53 | M | 36 | LL | 2.5 | ENL | AT | Y |
| ENL54 | M | 35 | LL | 4.25 | ENL | AD | Y |
| ENL55 | M | 61 | LL | 3.8 | ENL | DT | Y |
