# Supplementary material for: The Type I Interferon Pathway Is Upregulated in the Cutaneous Lesions and Blood of Multibacillary Leprosy Patients With Erythema Nodosum Leprosum
Source: Front Med (Lausanne). 2022 Jun 6;9:899998. doi: 10.3389/fmed.2022.899998 (PMC9208291; doi:10.3389/fmed.2022.899998)
Supplement: Supplementary file 4 [file Table_4.DOCX]

**Table S4 - Patients included in western blotting, immunohistochemistry, immunofluorescence and RT-qPCR follow ups of thalidomide treatment from skin lesions samples in figure 3.** M- Male. F- Female. NR – Non reactional, LL – Lepromatous Leprosy, ENL – Erythema Nodosum Leprosum, ENL_Thal_- ENL patient at 7^th^ day of thalidomide treatment. BI – Bacilloscopic Index. AD – At diagnostics; DT – During treatment; AT – After treatment. Y – Yes, N- No. IHC - Immunohistochemistry, IF - Immunofluorescence, WB - Western blotting

| **Patient ID** | **Sex** | **Age** | **Clinical Form** | **BI** | **Reaction type** | **Reaction diagnosis** | **First episode** | **Symbol** | **Type of analysis** |
| --- | --- | --- | --- | --- | --- | --- | --- | --- | --- |
| NR2 | M | 39 | LL | 5 | - | - | - |  | IF |
| NR42 | M | 17 | LL | 4.75 | - | - | - |  | WB |
| NR48 | M | 21 | LL | 4.5 | - | - | - |  | WB |
| NR49 | M | 38 | LL | 5 | - | - | - |  | IF |
| NR50 | F | 49 | LL | 5 | - | - | - |  | IF |
| NR69 | M | 47 | LL | 5.9 | - | - | - |  | WB |
| ENL1,ENL_Thal_1 | M | 40 | LL | 2.75 | ENL | AT | Y | **x** | RT-qPCR & IF |
| ENL2,ENL_Thal_2 | M | 34 | LL | 5.5 | ENL | AD | Y | ▲ | RT-qPCR & IF |
| ENL3,ENL_Thal_30 | M | 43 | LL | 3.75 | ENL | AT | Y |  | IF |
| ENL16, ENL_Thal_ 13 | M | 21 | LL | 2.5 | ENL | DT | N | ● | RT-qPCR, & WB |
| ENL17,ENL_Thal_14 | M | 27 | LL | 0 | ENL | AT | Y | 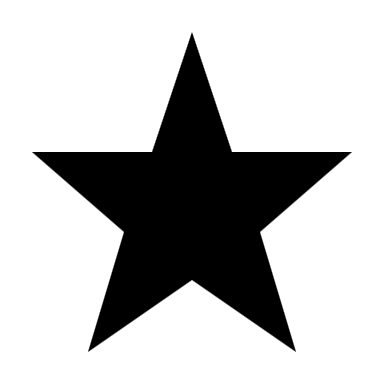 | RT-qPCR |
| ENL19,ENL_Thal_16 | M | 22 | LL | 4.5 | ENL | DT | Y | ♦ | RT-qPCR, & WB |
| ENL23,ENL_Thal_19 | M | 23 | LL | 4.57 | ENL | AT | Y | ▼ | RT-qPCR |
| ENL35,ENL_Thal_24 | M | 48 | LL | 4.5 | ENL | AT | Y | ■ | IHC; RT-qPCR &WB |
| ENL36,ENL_Thal_25 | M | 55 | LL | 2 | ENL | AT | N | **+** | RT-qPCR |
| ENL37,ENL_Thal_26 | M | 62 | LL | 2.6 | ENL | AT | Y |  | RT-qPCR |
| ENL49,ENL_Thal_31 | M | 42 | LL | 4 | ENL | AD | Y | ***** | RT-qPCR |
| ENL_Thal_ 3 | F | 50 | LL | 4.5 | ENL | DT | Y |  | IF |
