# Supplementary material for: The Type I Interferon Pathway Is Upregulated in the Cutaneous Lesions and Blood of Multibacillary Leprosy Patients With Erythema Nodosum Leprosum
Source: Front Med (Lausanne). 2022 Jun 6;9:899998. doi: 10.3389/fmed.2022.899998 (PMC9208291; doi:10.3389/fmed.2022.899998)
Supplement: Supplementary file 5 [file Table_5.DOCX]

**Table S5 - Patients included in plasmacytoid dendritic cells analysis in figure 5.** M- Male. F- Female. NR- Non reactional, LL – Lepromatous leprosy, BL – Borderline lepromatosum, ENL – Erythema Nodosum Leprosum. BI – Bacilloscopic Index. AD – At diagnostics; DT – During treatment; AT – After treatment. Y – Yes, N- No. FC- Flow Cytometry, IHC - Immunohistochemistry

| **Patient ID** | **Sex** | **Age** | **Clinical**  **Form** | **BI** | **Reaction** | **Reaction**  **diagnostics** | **First**  **episode** | **Type of analysis** |
| --- | --- | --- | --- | --- | --- | --- | --- | --- |
| NR7 | M | 54 | LL | 6 | - | - | - | FC |
| NR42 | M | 21 | LL | 4,75 | - | - | - | FC |
| NR70 | M | 35 | LL | 5 | - | - | - | FC |
| NR71 | M | 51 | LL | 5,75 | - | - | - | FC |
| NR72 | M | 36 | LL | 4 | - | - | - | FC |
| NR73 | M | 39 | LL | 5,75 | - | - | - | FC |
| NR74 | M | 17 | LL | 5,25 | - | - | - | IHC |
| NR75 | M | 48 | LL | 4,75 | - | - | - | IHC |
| NR76 | F | 78 | LL | 4,75 | - | - | - | IHC |
| NR77 | M | 48 | BL | 5 | - | - | - | IHC |
| ENL4 | M | 34 | LL | 4,5 | ENL | DT | Y | FC |
| ENL35 | M | 52 | LL | 5 | ENL | AT | N | FC & IHC |
| ENL37 | M | 62 | BL | 4,75 | ENL | AT | Y | IHC |
| ENL38 | M | 43 | LL | 4,25 | ENL | DT | Y | FC |
| ENL39 | M | 48 | LL | 5 | ENL | DT | N | FC |
| ENL51 | M | 38 | LL | 5 | ENL | AT | N | FC |
| ENL56 | M | 30 | BL | 2,75 | ENL | AT | Y | FC |
| ENL57 | F | 24 | LL | 5 | ENL | DT | Y | FC |
| ENL58 | M | 35 | LL | 3,25 | ENL | AD | Y | FC |
| ENL59 | F | 52 | LL | 5,5 | ENL | DT | Y | FC |
| ENL60 | F | 55 | LL | 4,5 | ENL | DT | Y | FC |
| ENL61 | M | 47 | LL | 5 | ENL | AT | Y | FC |
| ENL62 | M | 40 | LL | 5 | ENL | AT | N | FC |
| ENL64 | M | 62 | LL | 5 | ENL | AT | N | IHC |
| ENL65 | M | 50 | LL | 4,75 | ENL | AT | N | IHC |
