# Supplementary material for: The Type I Interferon Pathway Is Upregulated in the Cutaneous Lesions and Blood of Multibacillary Leprosy Patients With Erythema Nodosum Leprosum
Source: Front Med (Lausanne). 2022 Jun 6;9:899998. doi: 10.3389/fmed.2022.899998 (PMC9208291; doi:10.3389/fmed.2022.899998)
Supplement: Supplementary file 6 [file Table_6.DOCX]

**Table S6 – Oligonucleotide sequences used in RT-qPCR.**

| Gene | Sequence (5’- 3’) |
| --- | --- |
| RPL13a | Fw – 5’ GACAAGAAAAAGCGGATGGT 3’  Rv – 5’ GTACTTCCAGCCAACCTCGT 3’ |
| IFNb | Fw – 5’ GTCACTGTGCCTGGACCATA 3’  Rv – 5’ ACAGCATCTGCTGGTTGAAGA  3’ |
| IFNAR1 | Fw – 5’ TGCTGCGAAAGTCTTCTTGA 3’  Rv – 5’ CGATTTGTTCCTCAGAAGTTGA  3’ |
| IFI16 | Fw – 5’ GCTGGACCCAAAGGGAGTAA 3’  Rv – 5’ CTGTTTTCGGGTTCTCAGTTGAA  3’ |
| TBK1 | Fw – 5’ GAAGGGCGACGCTTAGTCTT 3’  Rv – 5’ ACCCCTGTTATTGCCTTAGCC  3’ |
| EIF2AK2 | Fw – 5’ TCTTCGTTGCTTATGAATGGTCT 3’  Rv – 5’ CCAAATCCACCTGAGCCAAT  3’ |
| MX1 | Fw – 5’ ACCTCGTGTTCCACCTGAAG 3’  Rv – 5’ TTCCTCCAGCAGATCCCTGA  3’ |
