# Supplementary material for: The Type I Interferon Pathway Is Upregulated in the Cutaneous Lesions and Blood of Multibacillary Leprosy Patients With Erythema Nodosum Leprosum
Source: Front Med (Lausanne). 2022 Jun 6;9:899998. doi: 10.3389/fmed.2022.899998 (PMC9208291; doi:10.3389/fmed.2022.899998)
Supplement: Supplementary file 7 [file Table_7.DOCX]

**Table S7 - DGE of type I Interferon pathway in ENL x NR.** lfcSE- standard error of log_2_Fold change. padj – adjusted p value.

| **SYMBOL** | **ENTREZID** | **GENENAME** | **baseMean** | **Log_2_FoldChange** | **lfcSE** | **pvalue** | **padj** |
| --- | --- | --- | --- | --- | --- | --- | --- |
| IFITM1 | 8519 | interferon induced transmembrane protein 1 | 6491,98 | 0,89 | 0,39 | 5,19884E-05 | 0,002896558 |
| GBP2 | 2634 | guanylate binding protein 2 | 5748,31 | 0,74 | 0,27 | 5,11926E-05 | 0,002891433 |
| IFITM2 | 10581 | interferon induced transmembrane protein 2 | 28460,86 | 0,70 | 0,29 | 0,000168523 | 0,006334672 |
| IRF1 | 3659 | interferon regulatory factor 1 | 7989,64 | 0,60 | 0,24 | 0,000411744 | 0,011143011 |
| HLA-F | 3134 | major histocompatibility complex, class I, F | 4079,97 | 0,58 | 0,17 | 3,25897E-05 | 0,002177181 |
| PSMB8 | 5696 | proteasome subunit beta 8 | 1882,63 | 0,57 | 0,18 | 7,11157E-05 | 0,003567425 |
| HLA-E | 3133 | major histocompatibility complex, class I, E | 43392,08 | 0,54 | 0,17 | 0,000105155 | 0,00458904 |
| IFITM3 | 10410 | interferon induced transmembrane protein 3 | 8404,68 | 0,54 | 0,35 | 0,002899927 | 0,034553993 |
| RNASEL | 6041 | ribonuclease L | 601,85 | 0,54 | 0,19 | 0,000241193 | 0,008135066 |
| HLA-C | 3107 | major histocompatibility complex, class I, C | 80040,57 | 0,51 | 0,21 | 0,000959394 | 0,018324683 |
| HLA-B | 3106 | major histocompatibility complex, class I, B | 81634,76 | 0,47 | 0,16 | 0,000469427 | 0,011858497 |
| IFI35 | 3430 | interferon induced protein 35 | 658,91 | 0,47 | 0,28 | 0,005878559 | 0,053703281 |
| IRF2 | 3660 | interferon regulatory factor 2 | 1459,45 | 0,44 | 0,18 | 0,001871179 | 0,027180098 |
| MYD88 | 4615 | MYD88 innate immune signal transduction adaptor | 3460,09 | 0,43 | 0,18 | 0,001935178 | 0,027843537 |
| IFNAR1 | 3454 | interferon alpha and beta receptor subunit 1 | 1483,22 | 0,43 | 0,22 | 0,006134514 | 0,055110844 |
| STAT1 | 6772 | signal transducer and activator of transcription 1 | 5598,27 | 0,42 | 0,23 | 0,008809783 | 0,068382545 |
| PTPN1 | 5770 | protein tyrosine phosphatase non-receptor type 1 | 858,14 | 0,39 | 0,17 | 0,004399661 | 0,04464912 |
| NLRC5 | 84166 | NLR family CARD domain containing 5 | 1476,80 | 0,39 | 0,17 | 0,005049241 | 0,048351968 |
| BST2 | 684 | bone marrow stromal cell antigen 2 | 949,91 | 0,36 | 0,22 | 0,019910274 | 0,111345555 |
| HLA-A | 3105 | major histocompatibility complex, class I, A | 38183,62 | 0,35 | 0,23 | 0,025374466 | 0,129359305 |
| IFNAR2 | 3455 | interferon alpha and beta receptor subunit 2 | 1200,42 | 0,34 | 0,17 | 0,011717012 | 0,081372052 |
| IRF9 | 10379 | interferon regulatory factor 9 | 2117,40 | 0,34 | 0,16 | 0,009495415 | 0,071560254 |
| PTPN6 | 5777 | protein tyrosine phosphatase non-receptor type 6 | 908,99 | 0,33 | 0,17 | 0,014446337 | 0,092017877 |
| IRF7 | 3665 | interferon regulatory factor 7 | 1052,04 | 0,30 | 0,23 | 0,051511863 | 0,196605218 |
| ADAR | 103 | adenosine deaminase RNA specific | 7211,30 | 0,30 | 0,16 | 0,023311354 | 0,122835664 |
| FADD | 8772 | Fas associated via death domain | 410,57 | 0,29 | 0,19 | 0,045386713 | 0,18270548 |
| ISG20 | 3669 | interferon stimulated exonuclease gene 20 | 1140,39 | 0,28 | 0,19 | 0,047276939 | 0,186775709 |
| ZBP1 | 81030 | Z-DNA binding protein 1 | 560,44 | 0,26 | 0,22 | 0,084836222 | 0,266158777 |
| YTHDF3 | 253943 | YTH N6-methyladenosine RNA binding protein 3 | 616,47 | 0,26 | 0,13 | 0,025452694 | 0,129471561 |
| UBE2K | 3093 | ubiquitin conjugating enzyme E2 K | 91,51 | 0,25 | 0,20 | 0,085527624 | 0,267255964 |
| PTPN2 | 5771 | protein tyrosine phosphatase non-receptor type 2 | 279,53 | 0,24 | 0,16 | 0,065784383 | 0,226581626 |
| HSP90AB1 | 3326 | heat shock protein 90 alpha family class B member 1 | 35,29 | 0,23 | 0,33 | 0,07954321 | 0,255991116 |
| TBK1 | 29110 | TANK binding kinase 1 | 89,68 | 0,23 | 0,25 | 0,126587765 | 0,336448601 |
| IFIT3 | 3437 | interferon induced protein with tetratricopeptide repeats 3 | 3349,09 | 0,22 | 0,26 | 0,134586341 | 0,348788099 |
| MUL1 | 79594 | mitochondrial E3 ubiquitin protein ligase 1 | 256,13 | 0,22 | 0,13 | 0,059889645 | 0,213936863 |
| STAT2 | 6773 | signal transducer and activator of transcription 2 | 2360,37 | 0,20 | 0,20 | 0,167220484 | 0,391434538 |
| MX2 | 4600 | MX dynamin like GTPase 2 | 3043,10 | 0,19 | 0,16 | 0,151018142 | 0,371769425 |
| TYK2 | 7297 | tyrosine kinase 2 | 2093,47 | 0,18 | 0,14 | 0,119756546 | 0,325303608 |
| CDC37 | 11140 | cell division cycle 37 | 1139,76 | 0,17 | 0,12 | 0,127068719 | 0,336937672 |
| SP100 | 6672 | SP100 nuclear antigen | 1621,82 | 0,15 | 0,15 | 0,219385068 | 0,45773415 |
| IRF3 | 3661 | interferon regulatory factor 3 | 650,40 | 0,15 | 0,18 | 0,281338181 | 0,526869004 |
| CNOT7 | 29883 | CCR4-NOT transcription complex subunit 7 | 474,45 | 0,15 | 0,15 | 0,240502409 | 0,482255191 |
| IFIT2 | 3433 | interferon induced protein with tetratricopeptide repeats 2 | 3200,50 | 0,14 | 0,22 | 0,352800056 | 0,597868428 |
| HLA-G | 3135 | major histocompatibility complex, class I, G | 5,85 | 0,13 | 0,24 | 0,362578181 | 0,606159432 |
| XAF1 | 54739 | XIAP associated factor 1 | 1450,32 | 0,12 | 0,24 | 0,412196985 | 0,647462526 |
| IFI27 | 3429 | interferon alpha inducible protein 27 | 290,95 | 0,11 | 0,34 | 0,241990565 | 0,483706609 |
| SAMHD1 | 25939 | SAM and HD domain containing deoxynucleoside triphosphate triphosphohydrolase 1 | 4014,31 | 0,09 | 0,17 | 0,483642045 | 0,703698518 |
| TRIM6 | 117854 | tripartite motif containing 6 | 22,23 | 0,07 | 0,25 | 0,594304998 | 0,781998103 |
| RSAD2 | 91543 | radical S-adenosyl methionine domain containing 2 | 877,51 | 0,02 | 0,28 | 0,850485466 | 0,930206814 |
| YTHDF2 | 51441 | YTH N6-methyladenosine RNA binding protein 2 | 416,40 | 0,01 | 0,10 | 0,903551002 | 0,955790256 |
| IP6K2 | 51447 | inositol hexakisphosphate kinase 2 | 130,93 | 0,00 | 0,15 | 0,998787352 | 0,99921267 |
| IRF6 | 3664 | interferon regulatory factor 6 | 5,02 | 0,00 | 0,27 | 0,977617713 | 0,989196704 |
| OASL | 8638 | 2'-5'-oligoadenylate synthetase like | 440,82 | -0,01 | 0,22 | 0,968216429 | 0,985440554 |
| IRF5 | 3663 | interferon regulatory factor 5 | 253,13 | -0,01 | 0,18 | 0,964060678 | 0,983376538 |
| IFI6 | 2537 | interferon alpha inducible protein 6 | 223,53 | -0,01 | 0,26 | 0,952920437 | 0,97761037 |
| JAK1 | 3716 | Janus kinase 1 | 2782,80 | -0,01 | 0,13 | 0,920491376 | 0,964343845 |
| IRAK1 | 3654 | interleukin 1 receptor associated kinase 1 | 744,44 | -0,03 | 0,08 | 0,738700385 | 0,87192009 |
| OAS1 | 4938 | 2'-5'-oligoadenylate synthetase 1 | 293,09 | -0,04 | 0,25 | 0,75064502 | 0,878974265 |
| LSM14A | 26065 | LSM14A mRNA processing body assembly factor | 980,63 | -0,05 | 0,12 | 0,608142056 | 0,791692642 |
| ISG15 | 9636 | ISG15 ubiquitin like modifier | 573,21 | -0,06 | 0,25 | 0,644977244 | 0,815055247 |
| TTLL12 | 23170 | tubulin tyrosine ligase like 12 | 240,61 | -0,06 | 0,12 | 0,547822127 | 0,749016747 |
| OAS3 | 4940 | 2'-5'-oligoadenylate synthetase 3 | 40,31 | -0,11 | 0,26 | 0,42489593 | 0,657879288 |
| MAVS | 57506 | mitochondrial antiviral signaling protein | 1058,92 | -0,11 | 0,09 | 0,193172229 | 0,425661364 |
| PTPN11 | 5781 | protein tyrosine phosphatase non-receptor type 11 | 361,50 | -0,12 | 0,10 | 0,212221754 | 0,450290043 |
| MX1 | 4599 | MX dynamin like GTPase 1 | 1592,41 | -0,13 | 0,24 | 0,377962418 | 0,619722955 |
| IRF8 | 3394 | interferon regulatory factor 8 | 22,62 | -0,15 | 0,29 | 0,260392751 | 0,503569166 |
| CACTIN | 58509 | cactin, spliceosome C complex subunit | 203,53 | -0,17 | 0,15 | 0,172836682 | 0,398610253 |
| IKBKE | 9641 | inhibitor of nuclear factor kappa B kinase subunit epsilon | 253,33 | -0,21 | 0,17 | 0,123096793 | 0,330604747 |
| OAS2 | 4939 | 2'-5'-oligoadenylate synthetase 2 | 1002,45 | -0,22 | 0,24 | 0,147634302 | 0,367742201 |
| IFIT1 | 3434 | interferon induced protein with tetratricopeptide repeats 1 | 595,14 | -0,23 | 0,29 | 0,104148411 | 0,299485107 |
| METTL3 | 56339 | methyltransferase like 3 | 215,54 | -0,24 | 0,13 | 0,034562162 | 0,155950138 |
| TREX1 | 11277 | three prime repair exonuclease 1 | 200,17 | -0,30 | 0,15 | 0,019459607 | 0,110029127 |
| USP18 | 11274 | ubiquitin specific peptidase 18 | 40,98 | -0,33 | 0,33 | 0,031932024 | 0,148680463 |
| ABCE1 | 6059 | ATP binding cassette subfamily E member 1 | 185,58 | -0,35 | 0,17 | 0,010796513 | 0,077331125 |
| IRF4 | 3662 | interferon regulatory factor 4 | 160,18 | -0,40 | 0,24 | 0,011841141 | 0,08190026 |
| EGR1 | 1958 | early growth response 1 | 26,14 | -0,46 | 0,25 | 0,00537939 | 0,050551921 |
