# Supplementary figures and images for: The Type I Interferon Pathway Is Upregulated in the Cutaneous Lesions and Blood of Multibacillary Leprosy Patients With Erythema Nodosum Leprosum
Source: Front Med (Lausanne). 2022 Jun 6;9:899998. doi: 10.3389/fmed.2022.899998 (PMC9208291; doi:10.3389/fmed.2022.899998)

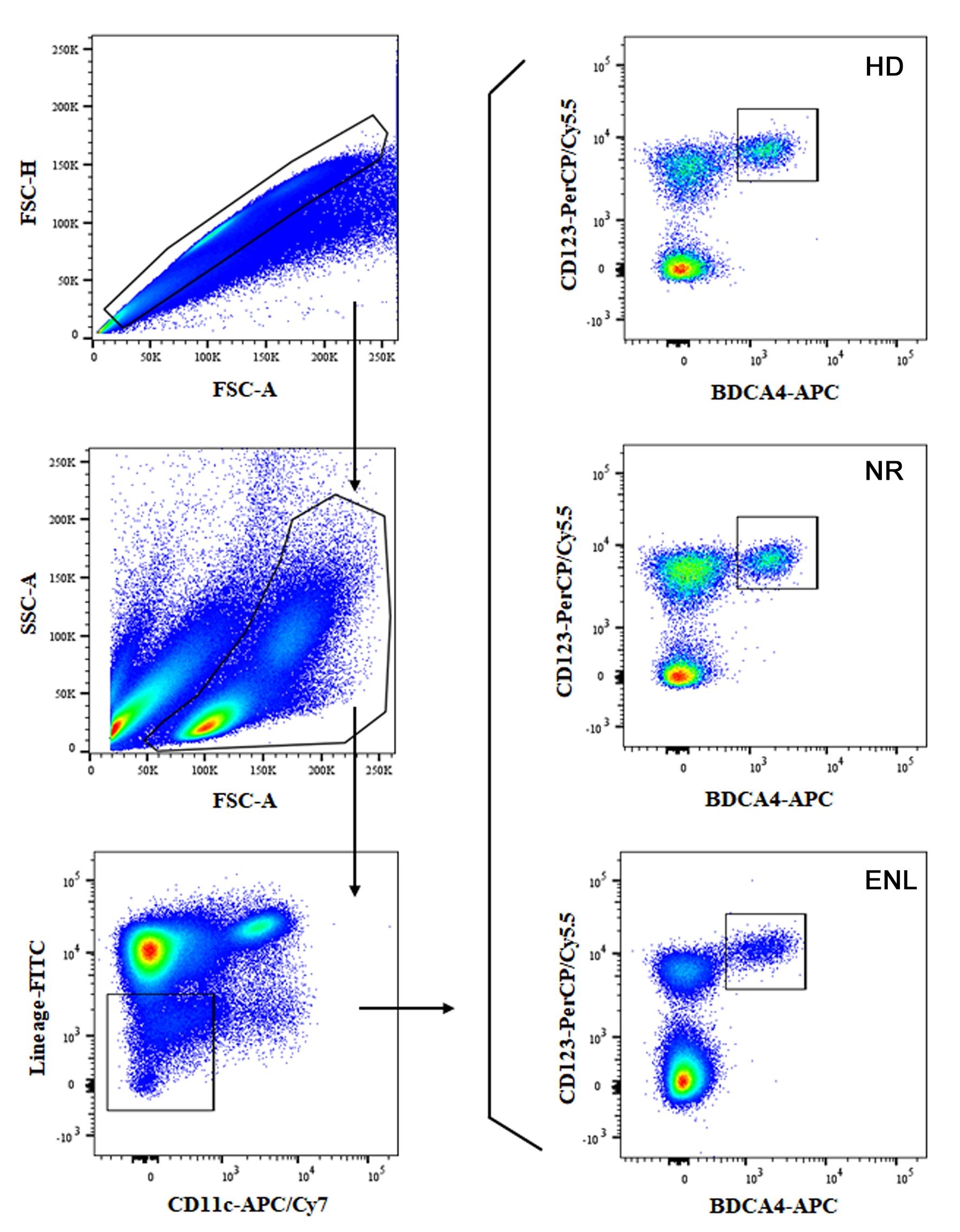

Supplement: Supplementary Figure 1 — Flow cytometry gating strategy to identify pDCs in PBMCs. Lumps and doublet cells were excluded using the parameters of frontal dispersion measured by area (FSC-A, forward scatter-area) versus frontal dispersion measured by height (FSC-H, forward scatter-height). Next, the PBMC region was selected by FSC-A and lateral dispersion was measured by SSC-A area (side scatter). In the region of PBMCs, pDCs were identified as lineages (CD3, CD14, CD19, CD20, and CD56)–, CD11c–, CD123+, and BDCA4+. Representative plots of pDCs for each study group are shown by using CD123 and BDCA4 markers. HD, healthy donor; NR, non-reactional multibacillary patient; and ENL, erythema nodosum leprosum. [file Image_1.TIF]

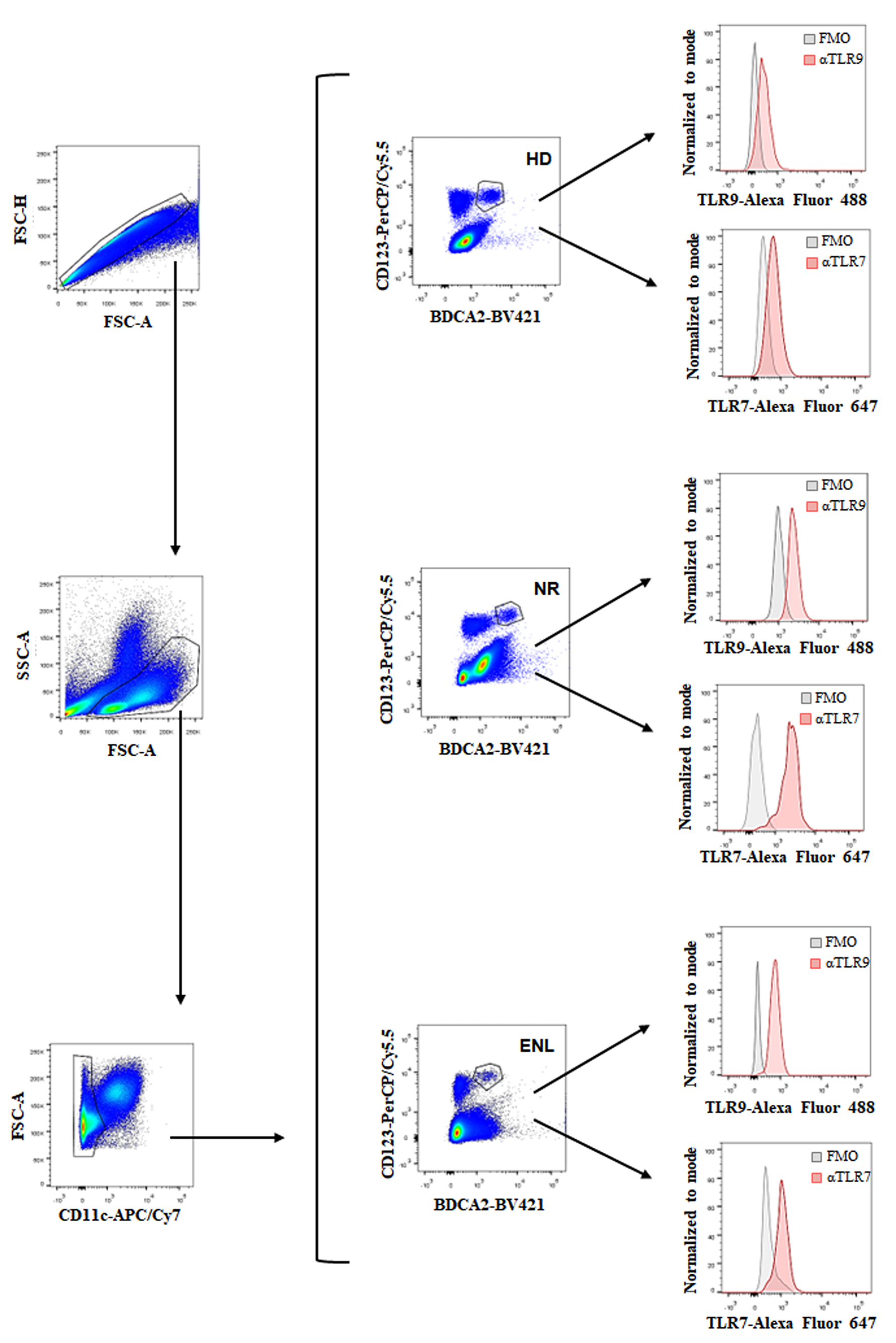

Supplement: Supplementary Figure 2 — Gating strategy and representative histograms of TLR9 and TLR7 levels in pDCs. Lumps and doublet cells were excluded using the parameters of frontal dispersion measured by area (FSC-A, forward scatter-area), versus frontal dispersion measured by height (FSC-H, forward scatter-height). The PBMC region was selected by FSC-A and lateral dispersion measured by SSC-A area (side scatter). In the region of PBMCs, pDCs were identified as CD11c–, CD123+ and BDCA2+. The expression levels of TLR9 and TLR7 by pDCs are shown in the representative histograms. HD, healthy donor; NR, non-reactional multibacillary patient, ENL, erythema nodosum leprosum; and FMO, fluorescence minus one. [file Image_2.TIF]
